# Smoking, alcohol consumption, and psoriasis risk: a systematic review and dose-response meta-analysis of observational studies

**DOI:** 10.3389/fpubh.2026.1840932

**Published:** 2026-06-05

**Authors:** Danping Chen, Guancheng Ye, Jie Yang, Lin Wang, Hailong Zhang, Zhihong Li

**Affiliations:** 1 Graduate School of Heilongjiang, University of Chinese Medicine, Harbin, China; 2 Department of Rheumatology, Dongzhimen Hospital, Beijing University of Chinese Medicine, Beijing, China; 3 The Fourth Affiliated Hospital of Heilongjiang, University of Chinese Medicine, Harbin, China; 4 The First Affiliated Hospital of Heilongjiang, University of Chinese Medicine, Harbin, China

**Keywords:** alcohol consumption, dose-response meta-analysis, incident psoriasis, psoriasis, smoking, systematic review

## Abstract

**Background:**

Smoking and alcohol consumption are modifiable behavioral exposures of public health relevance that may contribute to psoriasis risk, but the epidemiologic evidence remains inconsistent.

**Methods:**

We searched PubMed, Embase, Web of Science, and the Cochrane Library from inception to December 18, 2025, and included observational studies reporting adjusted associations between smoking or alcohol consumption and the risk of psoriasis. Random-effects meta-analyses and restricted cubic spline models were used where appropriate.

**Results:**

Thirty observational studies involving more than 25 million participants were included. Smoking was associated with a higher risk of psoriasis, with a summary relative estimate of 1.67 (95% CI: 1.46–1.90), and showed evidence of a positive dose-response relationship. Former smokers also remained at higher risk than never smokers (summary relative estimate = 1.38, 95% CI: 1.18–1.62). Alcohol consumption was associated with a smaller increase in psoriasis risk (summary relative estimate = 1.33, 95% CI: 1.16–1.53), and the estimate was further attenuated after trim-and-fill adjustment (summary relative estimate = 1.19, 95% CI: 1.02–1.37), indicating that the alcohol evidence is less robust and should be interpreted cautiously.

**Conclusions:**

Smoking shows a stronger and more consistent association with psoriasis risk, supported by dose-response evidence, whereas alcohol consumption shows a smaller and less consistent association. These findings highlight the public health relevance of smoking cessation and, with caution, alcohol reduction for psoriasis prevention, while also emphasizing the differences in evidence certainty between the two exposures.

**Systematic review registration:**

https://doi.org/10.37766/inplasy2026.1.0086, identifier: INPLASY202610086.

## 1 Introduction

Psoriasis is a chronic, immune-mediated inflammatory skin disease affecting approximately 2–3% of the global population (1, 2). Its clinical presentation is heterogeneous, including chronic plaque psoriasis and distinct phenotypes such as palmoplantar pustulosis (PPP) (1, 3). Beyond cutaneous manifestations, psoriasis is increasingly recognized as a systemic inflammatory disorder closely associated with psoriatic arthritis (PsA) and cardiometabolic comorbidities, thereby contributing substantially to long-term disease burden and morbidity. Although genetic susceptibility plays a central role in psoriasis development, environmental exposures and lifestyle factors may also be important contributors (1, 2).

Among potentially modifiable lifestyle factors, smoking and alcohol consumption have been widely investigated in relation to psoriasis risk (4–6). Several biological mechanisms may support these associations. Smoking may promote oxidative stress and inflammatory activation, including pathways involving reactive oxygen species and downstream cytokine signaling (1, 7). Nicotine may also contribute to abnormal angiogenesis through vascular endothelial growth factor (VEGF)-related mechanisms (1). Alcohol may impair skin barrier integrity and alter immune regulation, thereby potentially amplifying inflammatory cascades relevant to psoriasis development (8). From a public health perspective, clarifying the roles of smoking and alcohol consumption in psoriasis is important because both exposures are potentially modifiable and may inform prevention, health education, and risk communication strategies. However, epidemiologic findings across individual studies and previous reviews have remained inconsistent.

Although previous meta-analyses have examined these associations, important uncertainties remain. Earlier reviews did not consistently distinguish incident or newly diagnosed psoriasis from prevalent disease, provided limited evaluation of dose-response relationships across multiple exposure metrics, and gave relatively little attention to subtype-specific or progression-related outcomes such as PPP and PsA. In addition, methodological differences in smoking exposure definitions and comparator-group classification may have influenced the interpretation of pooled estimates (9). Several large population-based studies have also been published in recent years, warranting an updated synthesis of the available evidence.

To address these gaps, we conducted an updated systematic review and dose-response meta-analysis of smoking, alcohol consumption, and psoriasis risk, with the literature searched through December 18, 2025, in accordance with the MOOSE and PRISMA 2020 guidelines. Our objectives were to (1) evaluate the associations of smoking and alcohol consumption with incident psoriasis and selected subtype-specific outcomes; (2) examine dose-response relationships across available exposure metrics; and (3) explore the association between smoking cessation and subsequent psoriasis risk. A brief comparison between previous meta-analyses and the present study is presented in Table 1, with more detailed information provided in Supplementary Table 7.

**Table 1:** Comparison of previous meta-analyses and the present study.

Study	Exposure	Search end date	Included studies/designs	Dose-response	Smoking cessation	Subtype-specific outcomes	Key difference from the present study
Armstrong 2013	Smoking	15 Jun 2013	25 prevalence studies + 3 incidence studies	Limited/no formal pooled analysis	No	No formal pooled subtype analysis	Focused mainly on prevalence, with limited evidence on incident psoriasis
Zhou 2020	Smoking	12 Apr 2020	Cohort and case-control studies	No formal pooled analysis	Limited	PPP and PsA discussed separately	Smoking-focused review without integrated smoking-alcohol synthesis or detailed multi-metric dose-response analysis
Brenaut 2013	Alcohol	Up to Dec 2011	Heterogeneous observational studies	No	No	No	Systematic review only; no quantitative pooled meta-analysis for alcohol risk
Zhu 2012	Alcohol	Up to 2012	Case-control studies only	No	No	No	Early alcohol meta-analysis restricted to case-control studies and not focused on incident psoriasis
Present study	Smoking and alcohol	18 Dec 2025	Cohort, case-control, nested case-control, and twin studies	Yes	Yes(exploratory)	Yes (PPP, PsA)	Updated synthesis emphasizing incident/newly diagnosed psoriasis, with smoking dose-response analysis and exploratory analyses of cessation and subtype-specific outcomes

Differences in the number of included studies across reviews partly reflect differences in eligibility criteria, particularly the present review's emphasis on incident or newly diagnosed psoriasis where possible and exclusion of case–case analyses from the primary quantitative synthesis.

## 2 Methods

### 2.1 Protocol and registration

This systematic review and meta-analysis was conducted and reported in accordance with the Meta-analysis of Observational Studies in Epidemiology (MOOSE) guidelines (10) and the Preferred Reporting Items for Systematic Reviews and Meta-Analyses (PRISMA) 2020 statement (11). To enhance transparency and reduce the risk of bias, the study protocol was prospectively registered in INPLASY (International Platform of Registered Systematic Review and Meta-analysis Protocols; registration number: INPLASY202610086; doi: 10.37766/inplasy2026.1.0086). This review focused on modifiable behavioral exposures of potential public health relevance.

### 2.2 Search strategy

We conducted a comprehensive literature search in PubMed, Embase, Web of Science, and the Cochrane Library from inception toDecember18, 2025, to identify observational studies evaluating the associations of smoking and/or alcohol consumption with psoriasis risk. The search strategy combined Medical Subject Headings (MeSH)and free-text terms related to psoriasis, smoking or tobacco use, and alcohol consumption, without language restrictions. Detailed search strategies for all databases are provided in Supplementary Table 1. The search was updated prior to resubmission using the same databases, search terms, and eligibility criteria.

### 2.3 Eligibility criteria (PICOS)

#### 2.3.1 Inclusion criteria

##### 2.3.1.1 Population (P)

Studies were eligible if they involved the general population or individuals without the outcome of interest at baseline. For case-control studies, eligible studies included patients with psoriasis or selected psoriasis-related subtypes and control participants without the corresponding outcome. Studies addressing incident or newly diagnosed psoriasis were prioritized when available, in line with the primary etiologic focus of this review.

##### 2.3.1.2 Exposure/Intervention (E/I)

Eligible studies assessed smoking and/or alcohol consumption. Smoking exposure was categorized as current, former, or never smoking, with further classification by intensity, duration, or cumulative exposure when available. Alcohol exposure was categorized according to drinking status, frequency, or quantity of consumption, and was standardized to grams of ethanol per day whenever possible.

##### 2.3.1.3 Comparator (C)

Comparator groups included never smokers or those with the lowest smoking exposure, as well as non-drinkers or those with minimal alcohol consumption. In smoking cessation analyses, comparator groups were defined according to the specific contrast of interest (e. g, former vs current smokers or former vs never smokers).

##### 2.3.1.4 Outcome (O)

The primary outcome was psoriasis risk, with emphasis on incident or newly diagnosed disease whenever study design permitted. Selected subtype-specific outcomes, including palmoplantar pustulosis and psoriatic arthritis, were also extracted when reported. For psoriatic arthritis, studies conducted in the general population and studies evaluating progression from psoriasis to psoriatic arthritis were distinguished *a priori*; the latter were considered supplementary evidence rather than part of the primary incidence synthesis. Effect estimates were extracted as odds ratios (ORs), relative risks (RRs), or hazard ratios (HRs) with 95% Confidence Intervals, with preference given to the most fully adjusted multivariable estimates.

##### 2.3.1.5 Study design (S)

Eligible study designs included prospective and retrospective cohort studies, case-control studies, nested case-control studies, and twin studies. When both observational and Mendelian randomization analyses were reported in the same article, only the observational estimates were included in the quantitative synthesis, whereas Mendelian randomization findings were considered separately in the discussion.

#### 2.3.2 Exclusion criteria

Reviews, meta-analyses, editorials, conference abstracts, and case reports were excluded, as were animal studies and *in vitro* experiments. Studies were also excluded if effect estimates with corresponding confidence intervals were unavailable and could not be calculated. For duplicate publications from the same cohort, only the report with the largest sample size, longest follow-up, or most complete information was retained. Case-case analyses conducted exclusively among patients with psoriasis, such as comparisons of psoriatic arthritis vs. psoriasis without incident risk estimates, were excluded from the primary quantitative analyses and considered only narratively when relevant.

### 2.4 Study selection and data extraction

After duplicate removal using NoteExpress, two investigators (P.D.C. and J.Y.) independently screened titles and abstracts, and subsequently reviewed full texts according to predefined eligibility criteria. Disagreements were resolved through discussion with a third investigator (Z.H.L.).

Data were extracted using a standardized form, including: study characteristics (first author, publication year, country or region, study design, and follow-up duration); population characteristics (sample size, age, sex distribution, and number of cases and controls); exposure definitions and categorizations (smoking and alcohol exposure definitions, categories, and dose-related information for dose–response analyses); outcome definitions (methods of ascertaining psoriasis and related outcomes, including clinical diagnosis, self-reported physician diagnosis, or administrative database coding); and effect estimates (multivariable-adjusted ORs, RRs, or HRs with 95% confidence intervals), together with the covariates included in the adjusted models.

For each included study, we also extracted the covariates included in the most fully adjusted model, with particular attention to age/sex, BMI, smoking/alcohol mutual adjustment, socioeconomic factors, cardiometabolic variables, physical activity, and other study-specific confounders. These data were summarized in Supplementary Table 9.

To maintain etiologic consistency, we preferentially included studies addressing incident or newly diagnosed psoriasis and requiring extractable effect estimates for smoking and/or alcohol exposure.

### 2.5 Quality assessment of included studies

The methodological quality of the included studies was assessed using the Newcastle–Ottawa Scale (NOS), with separate versions for cohort and case–control studies (12). Based on the total NOS score, studies were classified as high quality (7–9 points), moderate quality (4–6 points), or low quality ( ≤ 3 points). Quality assessment was performed independently by two investigators, and disagreements were resolved through discussion. Detailed NOS scores for each included study are provided in Supplementary Table 3.

### 2.6 Data standardization for dose–response analyses

To improve comparability across studies, exposure metrics were standardized using a unified approach (13). Smoking exposure was expressed as cigarettes per day, smoking duration (years), or cumulative exposure in pack-years, whereas alcohol exposure was converted to daily ethanol intake in grams per day (g/day) whenever possible. If ethanol intake was not directly reported, conversion was based on study-defined standard drink units. When no study-specific definition was available, one standard drink was assumed to contain approximately 12.5 g of pure ethanol (14, 15). The potential impact of this assumption was considered in sensitivity analyses.

For studies reporting categorized exposure data, the median or mean value of each category was assigned as the representative dose. For open-ended categories (e.g., >20 cigarettes/day), the interval width was assumed to be the same as that of the adjacent category for dose assignment (16, 17). The reference group, defined as never smokers or non-drinkers, was assigned a value of zero exposure.

### 2.7 Statistical analysis

All statistical analyses were performed using Stata version 17.0 (StataCorp LLC, College Station, TX, USA). Effect estimates from the included studies, including odds ratios (ORs), relative risks (RRs), and hazard ratios (HRs), were log-transformed and pooled using random-effects models, yielding summary relative estimates with preference given to the most fully adjusted multivariable estimates. Because incident psoriasis was relatively uncommon in the included source populations, ORs and RRs were treated as approximately comparable on the relative-effect scale (18). HRs are time-to-event measures and are not directly equivalent to cumulative risk estimates. We did not convert all estimates to a single common metric because the included studies did not consistently report baseline risks, follow-up time distributions, or cumulative incidence information required for reliable conversion, particularly for HR-based studies. Moreover, converting adjusted ORs or HRs into RRs would have required additional assumptions that could introduce model-based uncertainty. Therefore, pooled estimates were interpreted as summary relative associations rather than exact interchangeable risk ratios.

For smoking, stratified analyses by effect measure type (OR-only, RR-only, HR-only) were conducted, and an HR-excluded sensitivity analysis was presented prominently in the Results to assess robustness across effect measures. For alcohol, formal stratification by effect measure type was limited by the small number of HR- and RR-based studies; therefore, only an OR-based supplementary analysis was presented (Supplementary Figure 9). Stratified analyses by effect measure type for smoking are presented in (Supplementary Table 8, Supplementary Figure 14).

Between-study heterogeneity was assessed using Cochran's *Q* test and the *I*^2^ statistic (19). Univariable meta-regression and subgroup analyses were used to explore heterogeneity according to geographic region and study design. Additional factors, including outcome ascertainment, exposure assessment, and adjustment characteristics, were considered where sufficiently and consistently reported across studies; however, formal quantitative exploration of these factors was limited by sparse and non-uniform reporting. For the primary smoking analysis, a 95% prediction interval was calculated based on the random-effects model to reflect the expected range of true effects in future comparable studies.

Leave-one-out sensitivity analyses were conducted to evaluate the influence of individual studies on the pooled estimates. Dose-response relationships were examined using a two-stage random-effects model with restricted cubic splines (RCS) to assess potential non-linear associations between smoking, alcohol consumption, and psoriasis risk. Knots were placed at the 10th, 50th, and 90th percentiles (20, 21). Non-linearity was assessed using a joint Wald test for the spline terms; when this test was not statistically significant (*P* < 0.05), a linear trend model was fitted.

Publication bias was evaluated by visual inspection of funnel plots and Egger's linear regression test (22). When statistically significant publication bias was detected (*P* < 0.05), the trim-and-fill method was considered where statistically appropriate and sufficiently supported by the number and structure of available studies (23). Smoking cessation-related analyses, including former vs. current smokers, former vs. never smokers, and time since cessation, were treated as exploratory because of the limited number and heterogeneity of available studies (23). All statistical tests were two-sided, and *P* < 0.05 was considered statistically significant (24, 25).

## 3 Results

### 3.1 Literature search and study selection

**Figure 1:**
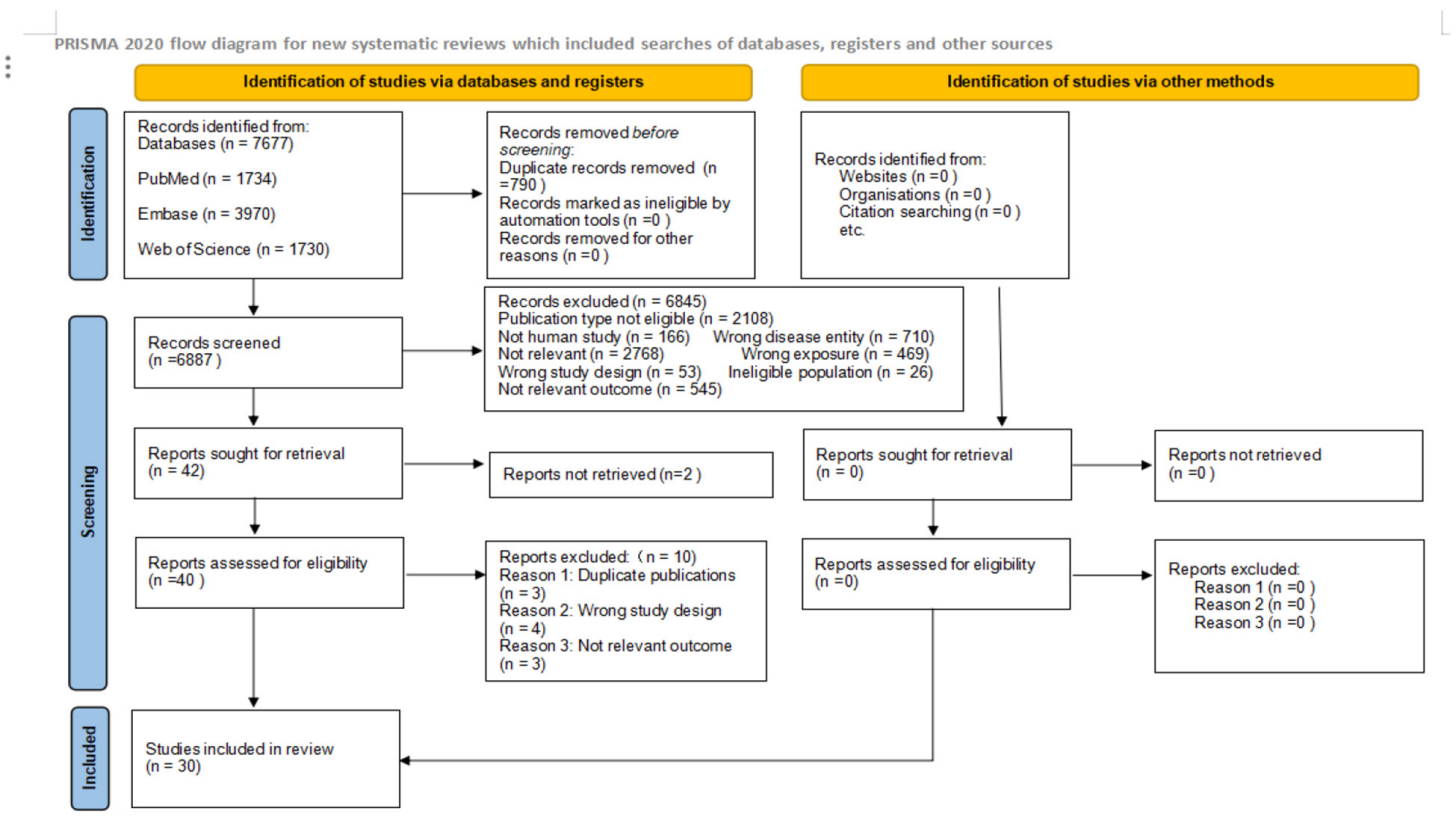
PRISMA 2020 flow diagram of the literature search and study selection process (1).

The initial search identified 7,677 records, including 1,734 from PubMed, 3,970 from Embase, 1,730 from Web of Science, and 243 from the Cochrane Library. After removal of 790 duplicates, 6,887 records remained for title and abstract screening. Following screening, 42 articles were retrieved for full-text review. Ultimately, 30 observational studies met the eligibility criteria and were included in the final analysis (Figure 1).

The literature search was updated through December 18, 2025; however, no additional studies published in 2025 met the eligibility criteria for quantitative inclusion. Records retrieved in 2025 were mainly excluded during title/abstract or full-text screening because of ineligible publication types (e.g., reviews, editorials, or conference abstracts), inappropriate study designs, non-eligible outcomes, or insufficient data for effect estimation. Compared with some earlier meta-analyses, the smaller number of included studies in the present review mainly reflected the stricter eligibility criteria, including the prioritization of incident or newly diagnosed psoriasis and the exclusion of case-case analyses from the primary quantitative synthesis.

The included studies comprised 17 cohort studies, 12 case-control studies, and one twin study (32). Published between 1990 and 2024, these studies were conducted in Asia (10 studies), Europe (13 studies), and North America (seven studies) (26–55). Detailed study characteristics are presented in Supplementary Table 2. The total sample size exceeded 25 million participants, largely driven by several nationwide administrative database cohort studies, including those by Lee EJ et al. in Korea (2017; approximately 17 million participants), Kim et al. in Korea (2024; approximately 5.78 million participants), and Taniguchi et al. in Japan (2024; approximately 710,000 participants). The twin study was retained as an observational study in the quantitative synthesis and was analyzed using the effect estimate reported in the original article. Given that only one twin study was included, its influence on the overall findings is unlikely to be substantial, although no separate exclusion analysis was prespecified.

### 3.2 Smoking exposure and psoriasis risk

#### 3.2.1 Risk of incident psoriasis

**Figure 2:**
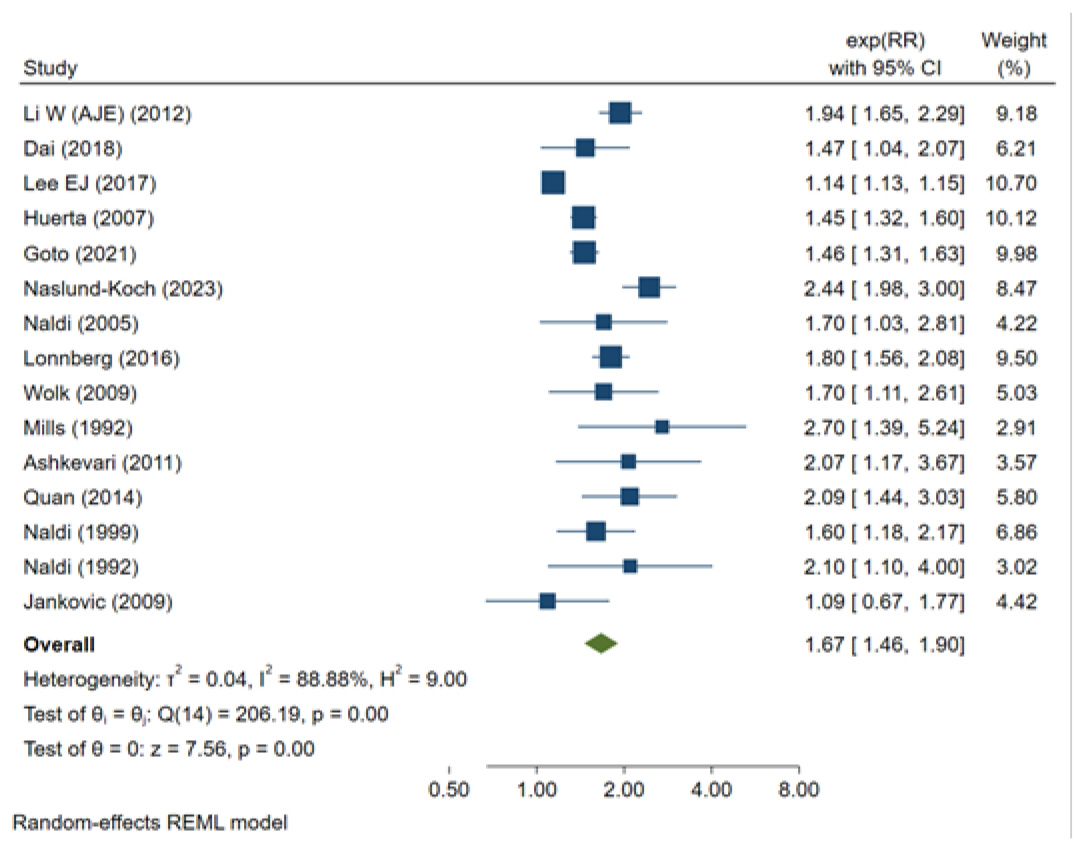
Forest plot of the overall meta-analysis examining the association between smoking and incident psoriasis risk. Effect estimates are presented as relative risks (RRs) with corresponding 95% confidence intervals (CIs). The pooled estimate was calculated using a random-effects model based on restricted maximum likelihood (REML). The diamond represents the overall combined effect.

Fifteen observational studies were included to evaluate the association between smoking and the risk of incident psoriasis, with a cumulative sample size of 17,948,978 individuals. The random-effects model yielded a summary relative estimate of 1.67 (95% CI: 1.46–1.90; *P* < 0.001), indicating a positive association between smoking and incident psoriasis (Figure 2). However, substantial between-study heterogeneity was observed (*I*^2^ = 88.9%). The 95% prediction interval was 1.06–2.63, suggesting that the magnitude of the association may vary considerably across future study settings. Therefore, this estimate should be interpreted as an average association across heterogeneous populations rather than as a precise setting-specific effect.

#### 3.2.2 Exploration of heterogeneity: meta-regression by geographic region

Given the substantial between-study heterogeneity in the overall analysis of smoking and incident psoriasis risk (*I*^2^ = 88.9%), a univariable random-effects meta-regression model using restricted maximum likelihood (REML) was conducted to explore potential sources of heterogeneity. Geographic region was included as a covariate, with Asia as the reference category and Europe and North America entered as comparison groups.

Meta-regression results suggested that geographic region explained only a limited proportion of the variability in effect estimates. Compared with studies conducted in Asia, the regression coefficient was 0.17 for studies from Europe (95% CI: −0.11 to 0.44; *P* = 0.232) and 0.27 for studies from North America (95% CI: −0.20 to 0.74; *P* = 0.259), with neither comparison reaching statistical significance. The overall Wald test also did not indicate a significant association between geographic region and effect size (Wald χ^2^ = 2.01; *P* = 0.3669).

This model explained approximately 10.5% of the between-study heterogeneity (*R*^2^ = 10.47%), whereas substantial residual heterogeneity remained (τ^2^ = 0.038; residual *I*^2^ = 80.7%). These findings suggest that additional factors, such as differences in smoking exposure definitions, outcome ascertainment, covariate adjustment, and population characteristics, may have contributed to the observed heterogeneity.

#### 3.2.3 Subgroup analysis by study design

To examine the consistency of the association across study designs, subgroup analyses were conducted according to study design. The results showed that the positive association between smoking and psoriasis risk was consistently observed across study types (Supplementary Figure 1). Among six cohort studies, the pooled relative risk was 1.59 (95% CI: 1.28–1.97), with substantial between-study heterogeneity (*I*^2^ = 96.3%). Among nine case-control studies, the pooled effect estimate was 1.78 (95% CI: 1.60–1.98), with very low between-study heterogeneity (*I*^2^ = 0.0%).

Although the level of heterogeneity differed between study designs, the confidence intervals for both pooled estimates did not cross the null value (RR = 1), supporting a generally consistent direction of association across study designs. Nevertheless, the magnitude of the effect should be interpreted cautiously in light of the substantial heterogeneity in cohort studies and the mixed effect measures included in the synthesis.

#### 3.2.4 Dose-response relationship

Nine studies provided stratified data on smoking dose. Dose-response analyses were conducted for cumulative exposure (pack-years), smoking duration, and smoking intensity (cigarettes per day). The main text prioritizes the pooled dose-response meta-analysis for cumulative smoking exposure, whereas additional study-level visualizations for smoking duration and smoking intensity are presented in Supplementary Figure 2.

**Figure 3:**
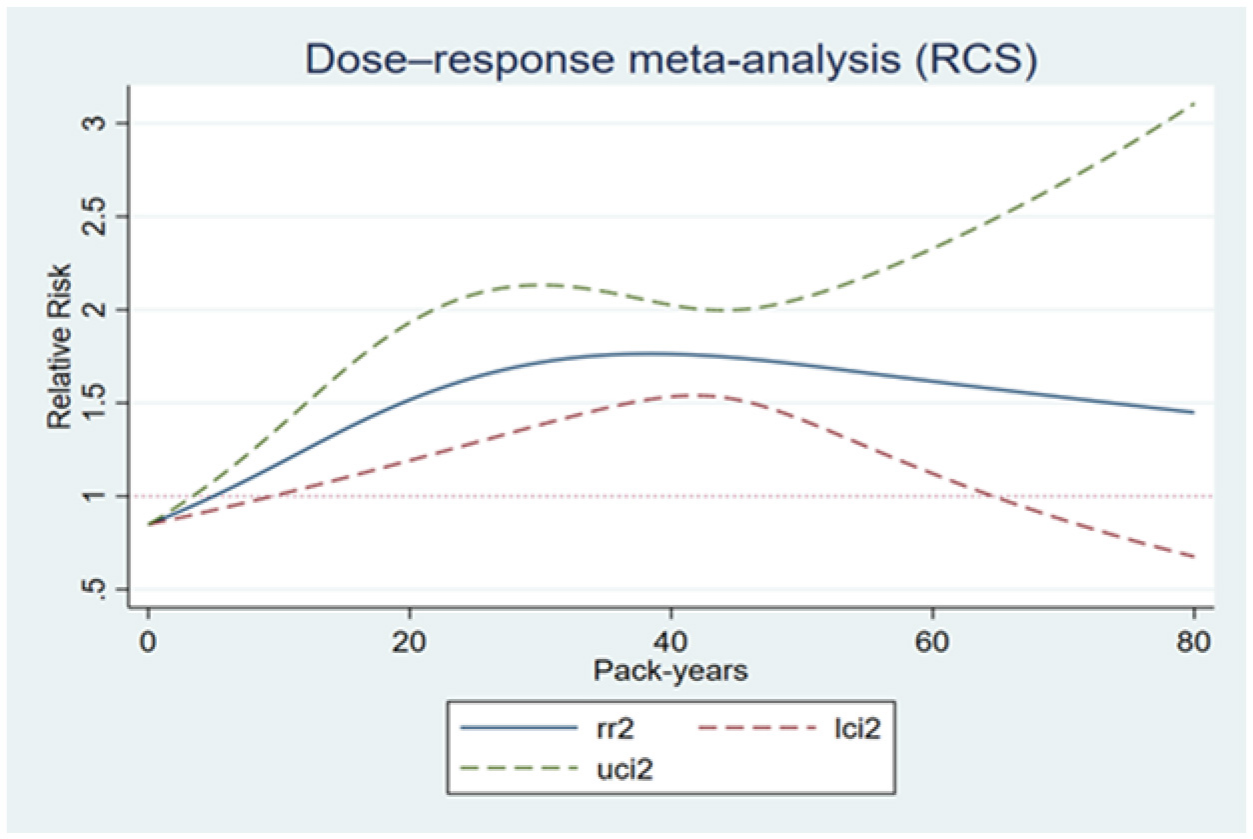
Dose-response relationship between cumulative smoking exposure and risk of psoriasis. A dose-response meta-analysis using restricted cubic splines was conducted to evaluate the association between cumulative smoking exposure (pack-years) and the risk of psoriasis. Solid lines represent pooled relative risks, and dashed lines indicate 95% confidence intervals. Detailed predicted values for selected exposure points are provided in Supplementary Table 6.

For cumulative smoking exposure measured in pack-years, restricted cubic spline (RCS) dose-response meta-analysis suggested a non-linear positive association between smoking and psoriasis risk (Figure 3). Compared with never smokers, the pooled relative risk was 1.25 (95% CI: 1.05–1.55) at 10 pack-years, 1.52 (95% CI: 1.20–1.95) at 20 pack-years, and 1.70 (95% CI: 1.35–2.15) at 30 pack-years. The estimated curve appeared to plateau at higher cumulative exposure levels, with predicted values for key exposure points presented in Supplementary Table 6.

Study-level visualizations for smoking intensity and smoking duration suggested that higher daily cigarette consumption and longer smoking history were generally associated with higher estimated psoriasis risk (Supplementary Figure 2). However, these descriptive plots should be interpreted cautiously because they were based on the available study-specific exposure data and do not represent pooled meta-analytic dose-response estimates.

#### 3.2.5 Subtype-specific effects and disease progression

Available evidence suggests that smoking may be differentially associated with psoriasis-related subtypes. For palmoplantar pustulosis (PPP), several studies suggested a positive association with smoking. For example, a large cohort study by Taniguchi et al. (54) reported that current smokers had a higher risk of PPP than non-smokers (HR = 2.47), and some studies also suggested stronger associations among heavier smokers. However, these findings should be interpreted as subtype-specific evidence and should not be regarded as directly equivalent to the primary analysis of incident psoriasis.

Five studies were included in the pooled analysis of psoriatic arthritis (PsA) and psoriasis-to-PsA progression outcomes (Supplementary Figure 4). The pooled relative risk was 1.95 (95% CI: 0.89–4.30; *P* = 0.09), but this estimate was imprecise and accompanied by extremely high between-study heterogeneity (*I*^2^ = 99.0%). The large variation in effect estimates across studies, including a modest association in Huo et al. (19) and a much stronger association in Naldi et al. (39), indicates that these findings should be regarded as exploratory. Accordingly, although the pooled estimate was above one, the current evidence is insufficient to support quantitative inference or definitive conclusions regarding smoking and PsA-related progression, and these findings should be considered hypothesis-generating only.

#### 3.2.6 Publication bias and effect-measure sensitivity analyses

In the overall analysis of smoking and psoriasis risk, Egger's regression test did not indicate statistically significant small-study effects (*P* = 0.1125; Supplementary Figure 3). Leave-one-out sensitivity analyses were also performed. Exclusion of any single study did not materially change the direction of the pooled estimate, suggesting that the overall positive association was not driven by any single study.

Additional stratified analyses by effect measure type (OR-only, RR-only, and HR-only) showed that pooled estimates for smoking were directionally consistent across effect measure types (Supplementary Table 8). To further evaluate the potential influence of combining different effect measures, we conducted a sensitivity analysis excluding HR-based estimates (Supplementary Figure 16). The HR-excluded sensitivity analysis yielded a summary relative estimate of 1.70 (95% CI: 1.36–2.12), compared with 1.67 (95% CI: 1.46–1.90) in the full primary analysis. The absolute difference between the two point estimates was 0.03, indicating that exclusion of HR-based estimates did not materially change the direction or approximate magnitude of the association. Nevertheless, substantial heterogeneity remained, suggesting that factors other than effect-measure type also contributed to between-study variability.

HRs were therefore retained in the primary synthesis for completeness, but they should still be interpreted cautiously because they are not fully equivalent to ORs or RRs.

### 3.3 Smoking cessation and risk reversal

#### 3.3.1 Former smokers vs. current smokers

This analysis included four primary studies contributing six effect estimates, with a total sample size of more than six million participants (*N* = 6,050,591). Overall, former smokers tended to show a lower risk than current smokers across several psoriasis-related outcomes (Supplementary Figure 5). Some studies suggested that this pattern might be more apparent for palmoplantar pustulosis (PPP) and generalized pustular psoriasis (GPP). However, several studies contributed multiple subtype-specific estimates, and the definitions of smoking cessation and outcome classification varied across studies. Accordingly, this analysis should be regarded as exploratory, and the pooled results were not intended to support quantitative inference regarding subtype-specific protective effects.

#### 3.3.2 Former smokers vs. never smokers

**Figure 4:**
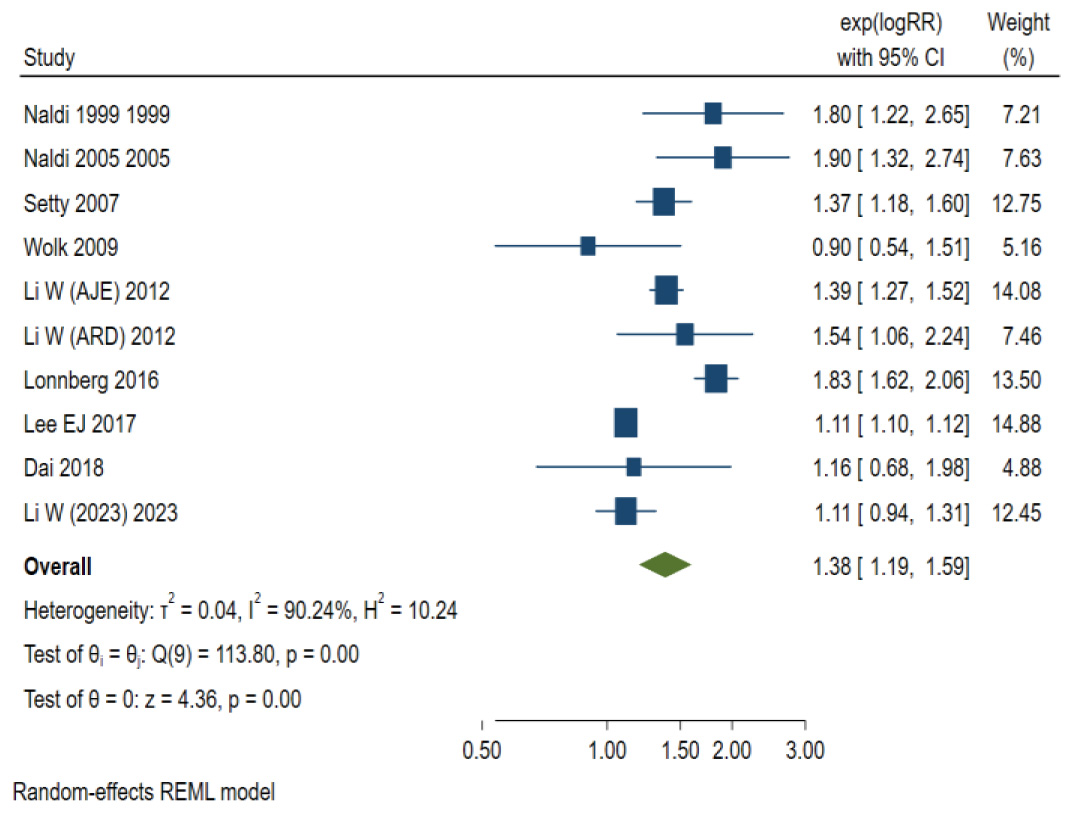
Residual risk of psoriasis in former smokers compared with never smokers. The forest plot presents relative risks (RRs) and corresponding 95% confidence intervals (CIs) for incident psoriasis in former smokers compared with never smokers across included studies. Pooled estimates were calculated using a random-effects model based on restricted maximum likelihood (REML). The diamond represents the overall combined effect.

Compared with never smokers, former smokers remained at a higher risk of psoriasis (RR = 1.38, 95% CI: 1.18–1.62; *N* = 10). This finding suggests that a history of smoking may be associated with residual excess risk after cessation, although the extent of risk reversal should be interpreted cautiously in light of the observational design and potential heterogeneity across studies (Figure 4).

#### 3.3.3 Time course of risk reduction after cessation

Trajectory analyses suggested that the relative risk among former smokers decreased with increasing time since smoking cessation. However, this analysis was based on data from only two cohort studies and should therefore be interpreted as descriptive and exploratory. The apparent decline in risk over time should be considered a hypothesis-generating pattern rather than a precise estimate of the time required for risk normalization. We therefore avoided drawing a definitive temporal threshold for risk recovery, and future prospective studies using standardized cessation intervals are needed to clarify the time course of psoriasis risk reduction after quitting.

#### 3.3.4 Publication bias and sensitivity analyses

In the analysis comparing former smokers with never smokers, Egger's regression test suggested potential small-study effects or publication bias (*P* = 0.035), indicating that the pooled estimate should be interpreted cautiously (Supplementary Figure 7). We did not apply the trim-and-fill method to the former-vs.-never smoker analysis because the number of studies was limited and the assumptions of trim-and-fill are unstable in the presence of between-study heterogeneity and correlated exposure categories. Leave-one-out sensitivity analyses were also performed to evaluate the influence of individual studies on the pooled result (Supplementary Figure 8). Exclusion of any single study did not materially change the overall direction of the association, suggesting that the observed residual risk was unlikely to be driven by any single study. Nevertheless, the possibility of publication bias should be considered when interpreting this finding.

### 3.4 Alcohol consumption and psoriasis risk

#### 3.4.1 Risk of incident psoriasis

**Figure 5:**
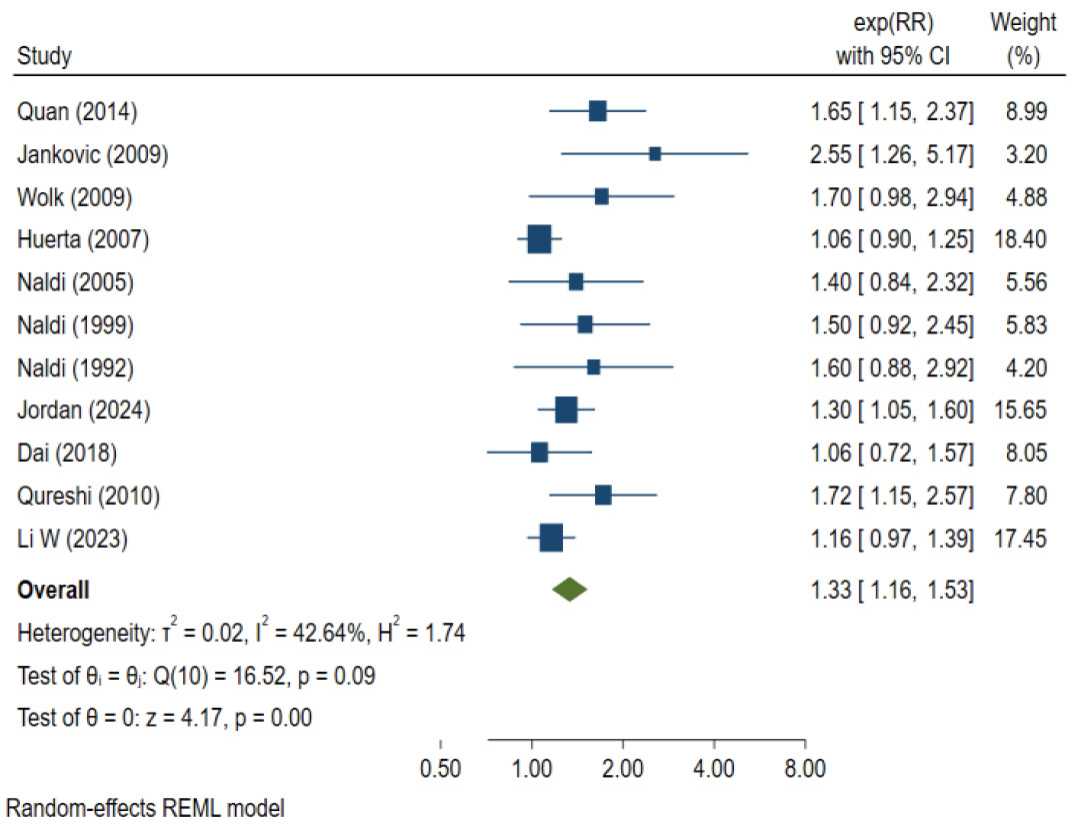
Forest plot of the overall meta-analysis examining the association between alcohol consumption and incident psoriasis risk. The forest plot presents relative risks (RRs) and corresponding 95% confidence intervals (CIs) for incident psoriasis associated with alcohol consumption across included studies. Pooled estimates were calculated using a random-effects model based on restricted maximum likelihood (REML). The diamond represents the overall combined effect.

Eleven observational studies were included to evaluate the association between alcohol consumption and the risk of incident psoriasis, with a combined sample size of 370,966 participants. Pooled analysis using a random-effects model showed that alcohol consumption was associated with a higher risk of psoriasis compared with non-drinkers, with a summary relative estimate of 1.33 (95% CI: 1.16–1.53; *P* < 0.001; Figure 5). Moderate between-study heterogeneity was observed (*I*^2^ = 42.6%). Because only a small number of alcohol-related studies reported HRs or RRs, formal stratified meta-analysis by effect measure type was not considered sufficiently informative; therefore, only an OR-based supplementary analysis was presented (Supplementary Figure 9). Covariate adjustment patterns, including whether alcohol-related analyses adjusted for smoking, are summarized in Supplementary Table 9.

#### 3.4.2 Publication bias assessment and trim-and-fill analysis

Visual inspection of the funnel plot suggested some asymmetry. Further assessment using Egger's regression test yielded a statistically significant result (*P* = 0.0013), indicating potential publication bias or small-study effects (Supplementary Figure 11).

To explore the possible influence of publication bias, a nonparametric trim-and-fill method was applied. This procedure imputed five potentially missing studies. Before adjustment, the summary relative estimate for the association between alcohol consumption and incident psoriasis was 1.33 (95% CI: 1.17–1.53); after trim-and-fill adjustment, the estimate was attenuated to 1.19 (95% CI: 1.02–1.37) (Supplementary Figure 12).

Although the magnitude of the association was reduced after trim-and-fill correction, the pooled estimate remained above one and statistically significant. These findings suggest that the observed positive association was not fully explained by potential publication bias, although the unadjusted pooled estimate may have overestimated the strength of the association and should therefore be interpreted with caution. The OR-based supplementary analysis for alcohol is presented in Supplementary Figure 9.

#### 3.4.3 Dose-response relationship

**Figure 6:**
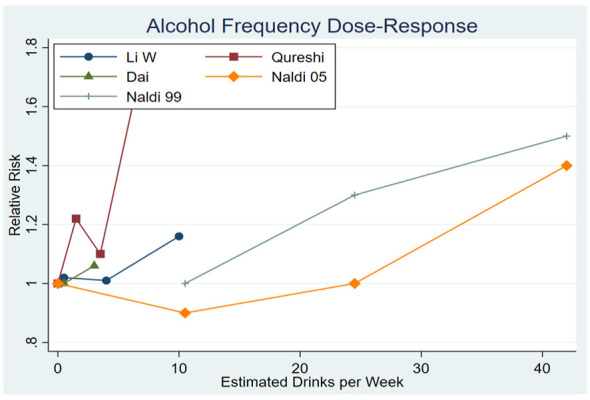
Dose-response relationship between alcohol drinking frequency and incident psoriasis. This figure presents the available dose-response evidence for incident psoriasis and is distinct from the single-study PsA spline shown in Supplementary Figure 10 and the descriptive study-level visualization shown in Supplementary Table 15.

Dose-response analyses were conducted for alcohol exposure in relation to psoriasis-related outcomes. For incident psoriasis, the available dose-response data suggested a tendency toward higher risk with greater drinking frequency (Figure 6).

For psoriatic arthritis (PsA), the corresponding restricted cubic spline analysis is presented separately in Supplementary Figure 10 because the available evidence is sparse and largely driven by a single study (62); accordingly, this curve should be interpreted as exploratory descriptive evidence rather than pooled dose-response meta-analytic evidence. In this analysis, higher alcohol intake was associated with higher estimated PsA risk, and the predicted curve suggested a non-linear increase at greater exposure levels. However, estimates at the upper end of alcohol intake were less stable because of sparse data, and the PsA findings should therefore be interpreted as exploratory rather than as definitive pooled meta-analytic evidence.

An additional study-level visualization of daily alcohol intake and psoriasis-related outcomes is provided in Supplementary Figure 15 for descriptive purposes.

#### 3.4.4 Progression from psoriasis to psoriatic arthritis

The secondary prevention analysis examined the association between alcohol consumption and progression from psoriasis to psoriatic arthritis (PsA). Three studies were included in the pooled analysis (Supplementary Figure 13), yielding a pooled relative risk of 2.80 (95% CI: 1.41–5.54; *P* = 0.003).

Wu et al. (62) reported an increased risk of PsA progression among heavier drinkers in a U.S. cohort, and Wang et al. (57) also observed a positive association in an Asian population. However, substantial between-study heterogeneity was present (*I*^2^ = 85.7%), indicating considerable variation in the magnitude of effect across studies.

Accordingly, although the pooled estimate was statistically significant, it was based on only three heterogeneous studies and should therefore be interpreted as limited and hypothesis-generating rather than definitive evidence for psoriasis-to-PsA progression. The current findings suggest that alcohol consumption may be relevant to psoriasis-to-PsA progression, but they are insufficient to support definitive conclusions regarding prevention strategies.

## 4 Discussion

### 4.1 Main findings

This study provides an updated synthesis of the associations of smoking and alcohol consumption with psoriasis risk. By integrating evidence from 30 observational studies involving more than 25 million participants, the present review extends previous evidence and provides an updated assessment of dose-response relationships.

The pooled analyses suggested that smoking was associated with a higher risk of incident psoriasis (pooled RR = 1.67, 95% CI: 1.46–1.90; *I*^2^ = 88.9%; 95% prediction interval: approximately 1.06–2.63), with dose-response analyses indicating increasing risk across multiple smoking exposure metrics, including smoking intensity, duration, and cumulative exposure. Given the broad prediction interval and substantial heterogeneity, this pooled estimate should be interpreted as an average association across heterogeneous study settings rather than as a precise setting-specific effect. These findings reflect the primary analysis, whereas the analyses of smoking cessation and subtype-specific outcomes, including psoriatic arthritis (PsA) and palmoplantar pustulosis (PPP), were based on more limited evidence and should therefore be interpreted cautiously.

For alcohol consumption, the pooled estimate also suggested a higher risk of psoriasis (RR = 1.33, 95% CI: 1.16–1.53). However, alcohol-related analyses showed clearer evidence of potential small-study effects (Egger's *P* = 0.0013), and after trim-and-fill adjustment, the pooled estimate was attenuated to RR = 1.19 (95% CI: 1.02–1.37), suggesting that the unadjusted association may have been overestimated, although a positive association remained. Alcohol-related dose-response findings, particularly for PsA, should also be interpreted cautiously because of the limited and heterogeneous evidence base. In addition, alcohol-related associations may be partly confounded by smoking and other behavioral or socioeconomic factors, particularly in studies without full mutual adjustment; covariate adjustment patterns across included studies are summarized in Supplementary Table 9.

Although both smoking and alcohol consumption were associated with higher psoriasis risk, the evidence for smoking was stronger and more consistent, supported by dose-response relationships across multiple metrics and a larger number of studies. In contrast, alcohol-related findings were based on fewer studies, showed a smaller effect size, and were more sensitive to publication bias and trim-and-fill adjustment. Therefore, while smoking cessation remains a clearly supported preventive target, the implications of alcohol reduction should be interpreted more cautiously.

Compared with previous meta-analyses, the present study evaluated both smoking and alcohol exposures, placed greater emphasis on incident or newly diagnosed psoriasis where feasible, and examined dose-response relationships across multiple smoking metrics. Smoking cessation and subtype-specific outcomes were treated as secondary or exploratory analyses rather than as evidence equivalent to the primary synthesis. A structured comparison is presented in Table 1, with more detailed information provided in Supplementary Table 7.

Stratified analyses by effect measure type indicated broadly consistent directions of association for smoking across studies reporting ORs, RRs, and HRs. However, because HRs are time-to-event measures and are not fully equivalent to cumulative risk estimates, these findings should be interpreted as supporting the overall direction of association rather than exact cross-measure comparability. Although ORs, RRs, and HRs are conceptually distinct, the HR-excluded sensitivity analysis yielded a similar estimate to the full primary analysis, supporting the robustness of the overall direction of association (Supplementary Figure 16). Nevertheless, pooled estimates combining different effect measures should be interpreted as summaries of the direction and approximate relative magnitude of association, rather than as exact cross-measure risk estimates. For alcohol-related analyses, formal stratification was limited by the small number of HR- and RR-based studies, and only an OR-based supplementary analysis was feasible (Supplementary Figure 9).

Substantial heterogeneity remained in the primary smoking analysis. Although geographic region explained only a small proportion of the between-study variability, other clinically relevant sources of heterogeneity, such as outcome ascertainment, exposure definition, and adjustment for major confounders including body mass index and mutual smoking/alcohol adjustment, could not be fully quantified because reporting was incomplete and non-uniform across studies. Because studies differed in exposure definitions and adjustment models.

In addition, alcohol-related associations may be partly confounded by smoking and other behavioral, metabolic, or socioeconomic factors, particularly in studies without full mutual adjustment. Because alcohol use often co-occurs with smoking, higher BMI, cardiometabolic risk, and socioeconomic disadvantage, the most likely direction of residual confounding would be away from the null, leading to potential overestimation of the alcohol–psoriasis association. This concern is particularly important because the alcohol effect size was modest and was attenuated after trim-and-fill adjustment; therefore, even moderate residual confounding could materially affect the alcohol estimate. Covariate adjustment patterns across included studies are summarized in Supplementary Table 9.

Overall, this study provides an updated synthesis of the associations of smoking and alcohol consumption with incident psoriasis while clarifying dose-response patterns and highlighting exploratory findings regarding smoking cessation and subtype-specific outcomes. The findings generally support the direction of previously reported associations, while also underscoring important limitations related to heterogeneity, small-study effects, and mixed effect measures. From a public health perspective, these results reinforce the relevance of smoking and alcohol consumption as potentially modifiable behavioral exposures that may contribute to psoriasis risk and may therefore be relevant to prevention-oriented counseling and risk communication.

### 4.2 Smoking and psoriasis: potential mechanisms and susceptibility to palmoplantar pustulosis

From a biological perspective, several mechanisms may help explain the observed association between smoking and psoriasis. The immunopathology of psoriasis involves keratinocyte-driven innate immune activation and amplification of the IL-23/Th17 axis (56, 57). In this context, polycyclic aromatic hydrocarbons and dioxin-like ligands in tobacco smoke may interfere with aryl hydrocarbon receptor (AhR) signaling. Because AhR is involved in skin barrier homeostasis, oxidative stress responses, and inflammatory regulation, sustained or dysregulated activation by exogenous ligands may contribute to a lower inflammatory threshold and chronic immune activation (58). These mechanisms are broadly compatible with the dose-response patterns observed in the present study, although mechanistic inference from epidemiologic data should remain cautious.

Subtype-specific findings in this review also suggested that smoking may have a stronger association with palmoplantar pustulosis (PPP) than with psoriasis overall. In some studies, the estimated risk for PPP was substantially higher than that observed for incident psoriasis in the primary analysis. This pattern may indicate that PPP is particularly susceptible to tobacco-related inflammatory triggers within the distinct anatomical and immunologic environment of the palms and soles (59). Mechanistically, PPP has been linked to neutrophil-predominant inflammation and IL-36/IL-17-related pathways, and smoking may plausibly enhance pustular inflammation through cytokine signaling at the epithelial-immune interface (60).

However, the PPP-related evidence in the present review was based on a limited number of studies, and heterogeneity in outcome definitions and exposure classification may affect the stability of the observed estimates. Accordingly, these findings should be regarded as hypothesis-generating rather than definitive, and further validation is needed (61).

### 4.3 Alcohol consumption and psoriasis: effect size, bias signals, and implications for psoriatic arthritis

Compared with smoking, the pooled effect size for alcohol consumption and psoriasis was smaller, although it remained statistically significant and was accompanied by lower between-study heterogeneity. Alcohol-related analyses showed clearer signs of potential small-study effects, as indicated by Egger's regression test (*P* = 0.0013). After trim-and-fill adjustment, the pooled effect estimate was attenuated from RR = 1.33 to RR = 1.19 (95% CI: 1.02–1.37), suggesting that the magnitude of the association may have been overestimated in the unadjusted analysis, although a positive association remained.

Residual confounding should also be considered when interpreting the alcohol-related findings. Smoking and alcohol consumption frequently co-occur, and incomplete adjustment for smoking may have inflated the observed alcohol–psoriasis association, particularly in studies that did not fully adjust for smoking exposure or used broad smoking categories. Residual confounding by BMI, cardiometabolic status, and socioeconomic position may also have biased the estimate in the same direction. This issue is particularly relevant because the alcohol effect size was modest and attenuated after trim-and-fill adjustment, meaning that even moderate residual confounding could materially influence the observed association. Therefore, alcohol-related findings should be interpreted as suggestive observational associations rather than evidence of an independent causal effect.

For alcohol-related analyses, interpretation across effect measure types was limited because only a small number of studies reported HRs or RRs. Therefore, we did not perform a formal pooled comparison analogous to that used for smoking; instead, we presented an OR-based supplementary analysis to provide a more homogeneous descriptive comparison. Accordingly, alcohol-related findings should not be interpreted as implying equivalence across ORs, RRs, and HRs.

From the perspective of disease spectrum, alcohol-related analyses also suggested possible relevance to psoriatic arthritis (PsA), particularly at higher levels of intake. However, the available PsA-related evidence was limited and heterogeneous, and the corresponding dose-response patterns should therefore be interpreted cautiously. Mechanistically, PsA has been linked to systemic inflammatory and immunometabolic pathways involving gut barrier function, the intestinal microbiota, and the gut-joint axis. Alcohol consumption may plausibly increase intestinal permeability and alter microbial balance, thereby contributing to systemic inflammation relevant to joint and entheseal disease (62, 63). Nevertheless, current evidence on alcohol and PsA remains limited by inconsistencies in outcome definitions, exposure stratification, and study design. Therefore, these observations should be regarded as hypothesis-generating and require further validation through prospective studies and triangulation across complementary lines of evidence (64, 65).

Additionally, most included studies focused on total alcohol intake rather than specific beverage types. Some reports suggested that certain beverages, such as non-light beer, might be more strongly associated with psoriasis risk, whereas light beer, red wine, and spirits did not show similarly clear associations (47). This pattern raises the possibility that factors beyond ethanol itself, such as beverage composition or related dietary exposures, may contribute to risk heterogeneity. At present, these findings are relevant mainly to hypothesis generation and are insufficient to support definitive recommendations regarding alcohol restriction specifically for PsA prevention.

### 4.4 Comparative patterns for smoking and alcohol consumption

A comparison of the available evidence suggests that both smoking and alcohol consumption are associated with a higher risk of psoriasis, although the evidence patterns differ between the two exposures. Smoking showed a larger pooled association and more apparent dose-response patterns across several exposure metrics, but was accompanied by substantial heterogeneity. In contrast, alcohol consumption showed a smaller pooled effect size and lower heterogeneity, but clearer signals of potential small-study effects, with attenuation of the pooled estimate after trim-and-fill adjustment.

These differences suggest that smoking and alcohol consumption may contribute to psoriasis risk through partly distinct biological and epidemiologic pathways, including more local cutaneous inflammatory effects for smoking and broader systemic inflammatory or immunometabolic effects for alcohol (59, 62). These contrasting patterns may also have implications for future risk stratification and lifestyle counseling. However, subtype-specific and progression-related findings in the present review remain limited, and any differential implications for palmoplantar pustulosis (PPP) or psoriatic arthritis (PsA) should be interpreted cautiously. At present, these observations are better regarded as hypothesis-generating rather than as a basis for established subtype-specific prevention strategies.

### 4.5 Smoking cessation, causal inference, and interpretation of Mendelian randomization findings

The observational meta-analytic findings of the present study do not fully align with the null or non-significant results reported in some Mendelian randomization (MR) analyses (e.g., Jordan 2024) (43). However, this discrepancy may reflect differences in exposure definitions, estimands, and underlying assumptions rather than a direct contradiction. MR uses genetic instrumental variables to estimate the effects of genetically proxied exposure and is less vulnerable to certain forms of confounding, but it may not adequately capture the complexity of real-world smoking behavior, including smoking intensity, duration, co-exposures, and socioeconomic context. In contrast, observational meta-analyses may better reflect disease patterns and exposure gradients in clinical and public health settings, while remaining more susceptible to residual confounding. Accordingly, these lines of evidence may be viewed as complementary, with observational findings informing risk stratification and hypothesis generation, and causal inference requiring triangulation across multiple methods.

The smoking cessation analyses in this review suggested that psoriasis risk among former smokers may gradually decrease with increasing time since cessation, although the available evidence was limited and the estimated recovery time should be interpreted cautiously (66). One possible explanation is that delayed risk reduction after cessation may reflect persistent biological changes related to prior smoking exposure. Emerging evidence has linked psoriasis recurrence to local tissue immune memory and phenotypic reprogramming of keratinocytes, which may contribute to a lower threshold for disease reactivation (67). In addition, smoking-related DNA methylation changes may reverse only gradually after cessation, potentially providing a molecular context for the prolonged decline in risk (68, 69). These mechanistic considerations are biologically plausible but do not establish causality for the epidemiologic pattern observed in this study. Future prospective cohorts should use standardized cessation intervals to clarify the time course of psoriasis risk reduction after quitting.

### 4.6 Strengths and limitations

This study has several strengths. First, it included a large overall sample size, which improved the precision of pooled estimates and allowed an updated synthesis of the available evidence on smoking, alcohol consumption, and psoriasis risk. Second, the study incorporated dose-response analyses across multiple smoking exposure metrics and additionally considered smoking cessation and selected subtype-specific outcomes such as palmoplantar pustulosis (PPP) and psoriatic arthritis (PsA), although these latter analyses were based on more limited evidence. Third, we conducted several assessments of robustness and potential bias. No statistically significant small-study effects were detected in the overall smoking analysis (Egger's *P* = 0.1125), whereas alcohol-related analyses showed evidence of possible small-study effects and were therefore further examined using trim-and-fill adjustment. Finally, the overall methodological quality of the included studies was generally moderate to high, with most studies achieving Newcastle-Ottawa Scale (NOS) scores of at least seven.

Several limitations should also be acknowledged. First, substantial between-study heterogeneity remained, particularly in the primary smoking analysis. Although heterogeneity was explored, univariable meta-regression showed that geographic region explained only a limited proportion of the observed variability (Wald χ^2^ = 2.01, *P* = 0.3669; *R*^2^ ≈ 10.47%). Additional clinically relevant sources of heterogeneity likely included differences in outcome ascertainment, exposure assessment, and adjustment for major confounders such as body mass index, as well as mutual adjustment for smoking and alcohol exposure. More detailed quantitative exploration of these factors was limited by incomplete and non-uniform reporting across the included studies. Therefore, the pooled estimates should be interpreted as summaries of direction and overall magnitude rather than as precise effect sizes applicable across all study settings.

Second, although ORs and RRs were considered approximately comparable under a low-incidence assumption, HRs are conceptually distinct time-to-event measures and may not be fully equivalent to cumulative risk estimates. We therefore performed additional stratified and sensitivity analyses for smoking and a limited alcohol-related supplementary analysis restricted to OR-based studies; however, some cross-measure comparability concerns remain. Accordingly, pooled estimates involving different effect measures should be interpreted with caution despite the supporting sensitivity analyses.

Third, smoking and alcohol consumption are closely related to socioeconomic status, body mass index, cardiometabolic status, and psychological or behavioral factors, and residual confounding cannot be excluded despite multivariable adjustment in most included studies. The most plausible direction of residual confounding is overestimation of the observed associations, especially for alcohol, because alcohol consumption frequently co-occurs with smoking and other psoriasis-related risk factors. Given the modest alcohol effect size and attenuation after trim-and-fill adjustment, residual confounding could have a meaningful influence on the alcohol estimate. However, the exact magnitude of this bias could not be quantified because the included studies differed in exposure definitions, adjustment models, and reporting detail. Covariate adjustment patterns are summarized in Supplementary Table 9.

Fourth, although the literature search was updated through December 18, 2025, no eligible studies published in 2025 met the inclusion criteria; thus, the quantitative synthesis ultimately reflected the available evidence through 2024. Fifth, we did not systematically search trial registries or other gray literature sources, which may have increased the possibility of publication or reporting bias. Finally, publication bias or small-study effects were suggested in some alcohol-related and residual-risk analyses, indicating that those findings should be interpreted cautiously.

## 5 Conclusions

This systematic review and meta-analysis suggests that smoking is associated with a higher risk of psoriasis, with supportive dose-response evidence across several exposure metrics. Alcohol consumption was also associated with a modestly higher psoriasis risk, but this association was less robust, attenuated after adjustment for potential publication bias, and may be more susceptible to residual confounding. Therefore, smoking cessation is more strongly supported as a prevention-oriented public health target, whereas implications regarding alcohol reduction should be interpreted more cautiously. Subtype-specific findings, particularly those related to PPP and PsA, remain limited and hypothesis-generating. Overall, causal inference and subtype-specific preventive implications remain uncertain and require confirmation in future well-designed prospective studies.

## Data Availability

Publicly available datasets were analyzed in this study. This data can be found here: the data analyzed in this study are derived from previously published studies and are available in the referenced articles.
