# Supplementary material for: Smoking, alcohol consumption, and psoriasis risk: a systematic review and dose-response meta-analysis of observational studies
Source: Front Public Health. 2026 Jun 5;14:1840932. doi: 10.3389/fpubh.2026.1840932 (PMC13279607; doi:10.3389/fpubh.2026.1840932)
Supplement: Supplementary file 1 [file Data_Sheet_1.docx]

Supplementary Material

Contents

[**Supplementary Tables** 4](#_Toc229413226)

[Supplementary Table S1. Detailed search strategies and results for each database Search Date: December 18, 2025 4](#_Toc229413227)

[Supplementary Table S2. Characteristics of studies included in the meta-analysis 5](#_Toc229413228)

[Supplementary Table S3. Quality assessment of included studies 8](#_Toc229413229)

[Supplementary Table S4. Exposure assignment and standardization methods used in dose–response analyses 9](#_Toc229413230)

[Supplementary Table S5. PRISMA 2020 Checklist 12](#_Toc229413231)

[Supplementary Table S6. Predicted Relative Risks of Psoriasis by Cumulative Smoking Exposure 14](#_Toc229413232)

[Supplementary Table S7. Detailed comparison of previous meta-analyses and the present study 15](#_Toc229413233)

[Supplementary Table S8. Stratified analyses by effect measure type for the association between smoking and incident psoriasis 17](#_Toc229413234)

[Supplementary Table S9. Covariate adjustment for mutual smoking/alcohol confounding and other key covariates 17](#_Toc229413235)

[**Supplementary Figures** 27](#_Toc229413236)

[Supplementary Figure S1. Association between smoking and incident psoriasis stratified by study design 27](#_Toc229413237)

[Supplementary Figure S2. Study-level dose–response visualizations for smoking exposure and incident psoriasis risk 27](#_Toc229413238)

[Supplementary Figure S3. Funnel plot assessing publication bias for the association between smoking and psoriasis risk 27](#_Toc229413239)

[Supplementary Figure S4. Association between smoking and psoriasis-related outcomes including disease incidence and progression 28](#_Toc229413240)

[Supplementary Figure S5. Smoking cessation and psoriasis risk relative to current smoking across clinical subtypes 29](#_Toc229413241)

[Supplementary Figure S6. Long-term trajectory of psoriasis risk following smoking cessation 29](#_Toc229413242)

[Supplementary Figure S7. Funnel plot assessing publication bias for residual psoriasis risk in former smokers 29](#_Toc229413243)

[Supplementary Figure S8. Leave-one-out sensitivity analysis of residual psoriasis risk in former smokers 30](#_Toc229413244)

[Supplementary Figure S9. Association between alcohol consumption and incident psoriasis based on OR-only studies 31](#_Toc229413245)

[Supplementary Figure S10. Dose–response relationship between alcohol intake and risk of psoriatic arthritis 31](#_Toc229413246)

[Supplementary Figure S11. Funnel plot assessing publication bias for the association between alcohol consumption and incident psoriasis 31](#_Toc229413247)

[Supplementary Figure S12. Trim-and-fill analysis for the association between alcohol consumption and incident psoriasis 32](#_Toc229413248)

[Supplementary Figure S13. Association between alcohol consumption and risk of psoriatic arthritis (secondary prevention analysis) 32](#_Toc229413249)

[Supplementary Figure S14. Stratified analysis of the association between smoking and incident psoriasis by effect measure type 33](#_Toc229413250)

[Supplementary Figure S15. Study-level dose–response visualization of daily alcohol intake and psoriasis-related outcomes 34](#_Toc229413251)

[Supplementary Figure S16. Sensitivity analysis excluding HR-based estimates for the association between smoking and incident psoriasis 34](#_Toc229413252)

**Supplementary Tables**

**Supplementary Table S1. Detailed search strategies and results for each database Search Date: December 18, 2025 1**

| Database | Search Query | | Results (Hits) |
| --- | --- | --- | --- |
| PubMed | | ("Psoriasis"[Mesh] OR psoriasis[Title/Abstract] OR psoriatic[Title/Abstract] OR psoriases[Title/Abstract]) AND (("Smoking"[Mesh] OR smoking[Title/Abstract] OR smoker[Title/Abstract] OR cigarette[Title/Abstract] OR tobacco[Title/Abstract] OR nicotine[Title/Abstract]) OR ("Alcohol Drinking"[Mesh] OR "Alcoholic Beverages"[Mesh] OR alcohol[Title/Abstract] OR drinking[Title/Abstract] OR "alcohol consumption"[Title/Abstract] OR ethanol[Title/Abstract])) | [1734] |
| Embase | | (psoriasis OR psoriases OR psoriatic) AND (smoking OR smoker OR cigarette OR cigarettes OR tobacco OR nicotine OR alcohol OR drinking OR "alcohol consumption" OR ethanol) | [3970] |
| Web of Science | | TS=(psoriasis OR psoriases OR psoriatic) AND (smoking OR smoker OR cigarette OR cigarettes OR tobacco OR nicotine OR alcohol OR drinking OR "alcohol consumption" OR ethanol) | [1730] |
| Cochrane Library | | (psoriasis OR psoriases OR psoriatic) AND (smoking OR smoker OR cigarette OR cigarettes OR tobacco OR nicotine OR alcohol OR drinking OR "alcohol consumption" OR ethanol) | [243] |

Note: MeSH, Medical Subject Headings; TS, Topic Search (includes title, abstract, author keywords, and Keywords Plus).

**Supplementary Table S2. Characteristics of studies included in the meta-analysis 1**

| Study | Country | Region | Design | Sample Size (N) | Exposure Metrics | Outcome Definition | Effect Measure (95% CI) | Key Adjustments |
| --- | --- | --- | --- | --- | --- | --- | --- | --- |
| Kim et al. (2024) | Korea | Asia | Retrospective Cohort | 5,784,973 | Smoking cessation status; pack-years | Incident PsV, PPP, and GPP | aHR: 0.91 (0.87–0.95) | Age BMI, alcohol, income |
| Taniguchi et al. (2024) | Japan | Asia | Retrospective Cohort | 717,472 | Smoking status; daily alcohol use | Incident PPP (ICD-10 L40.3) | HR: Direction reported | Age, sex, BMI, tonsillitis |
| Goto et al. (2021) | Japan | Asia | Prospective Cohort | 487,835 | Smoking status; BMI; GGT levels | Incident late-onset PsO | HR (Smoking): 1.46 (1.31–1.63) | Exercise, weight gain, comorbidities |
| Wang et al. (2021) | China | Asia | Case-Control | 1,539 | Smoking and alcohol habits | PsO and PsA (CASPAR) | Adjusted OR (Smoking): 1.43 | Education, BMI, comorbidities |
| Dai et al. (2019) | Taiwan | Asia | Retrospective Cohort | 60,136 | Smoking (cigs/d, pack-yrs); Alcohol | Incident PsO (ICD-9-CM codes) | aHR (Current): 1.47 (1.04–2.07) | Age, BMI, marital status |
| Lee EJ et al. (2017) | Korea | Asia | Nationwide Cohort | 17,055,608 | Smoking status; intensity; duration | Incident PsO cases | Adj IR (Current): 1.14 (1.13–1.15) | BMI, alcohol, exercise |
| Quan et al. (2014) | China | Asia | Case-Control | 660 | Smoking/Alcohol (Yes/No); BMI | Clinically diagnosed PsV | OR (Smoke): 2.09 (1.44–3.03) | Age, sex, BMI, smoking |
| Zheng et al. (2004) | China | Asia | Case-Control | 522 | Smoking and alcohol habits | Clinically diagnosed PsO | OR: Exact extractable estimate not available | Sex and HLA genotypes |
| Zhang et al. (2002) | China | Asia | Case-Control | 1,578 | Smoking status; cigs/day; alcohol | Clinically diagnosed PsO (BSA) | OR (Smoking): Positive link | Stratified by sex |
| Ashkevari et al. (2011) | Iran | Asia | Case-Control | 192 | Smoking status; pack-years | Psoriasis Vulgaris (PV) | OR (Smoke >5 pack-yrs): 2.07 | Matched by age, sex, and BMI |
| Jordan et al. (2024) | Denmark | Europe | Cohort + MR | 102,655 | Alcohol (g/wk); Genotypes | Registry-based PsO (ICD codes) | HR: 1.30 (1.05–1.60); MR-OR: 0.93 | Smoking, BMI, SES |
| Näslund-Koch (2023) | Denmark | Europe | Cohort + MR | 105,912 | Smoking (pack-yrs); CHRNA3 | Registry-based moderate-to-severe PsO | HR (obs): 2.44 (1.98–3.00); MR: NS | Age, alcohol, BMI, lipids |
| Green et al. (2020) | UK | Europe | Prospective Cohort | 90,189 | Smoking; Alcohol units/day; BMI | Incident PsA progression | OR(Alcohol≥2 units/d): 1.57 | Age, PsO severity, BMI |
| Lønnberg et al. (2016) | Denmark | Europe | Twin Cohort Study | 34,781 | Smoking status; pack-years; ETS | Self-reported physician diagnosis | OR (Smoking >5 pack-yrs): 2.18 | Age, childhood ETS exposure |
| Jankovic et al. (2009) | Montenegro | Europe | Case-Control | 310 | Smoking status; intensity; alcohol | Clinically diagnosed PsO | OR (Alcohol): 2.55 (1.26–5.17) | Sex, age, BMI, stress |
| Wolk et al. (2009) | Sweden | Europe | Case-Control | 746 | BMI; smoking status; alcohol | Incident plaque PsO | OR(Current smokers): Significant risk | SES, weight gain, alcohol |
| Huerta et al. (2007) | UK | Europe | Nested Case-Control | 13,994 | Smoking and alcohol (GP records) | Incident PsO (GP diagnosis codes) | Adjusted OR (Smoking): 1.45 | GP visits, BMI, age |
| Naldi et al. (2005) | Italy | Europe | Case-Control | 1,250 | Smoking status; intensity; BMI | Dermatologist-diagnosed incident PsO | OR (BMI≥30): 1.9 (1.2–2.8) | Age, sex, education |
| Naldi et al. (1999) | Italy | Europe | Case-Control | 1,020 | Smoking; alcohol; sex-specific | Dermatologist-diagnosed incident PsO | OR (Current smoker): 1.7 (1.1–2.7) | BMI, family hx, alcohol |
| Poikolainen (1994) | Finland | Europe | Case-Control | 163 | Cigs/day; alcohol; life events | Clinically diagnosed PsO | OR (20 cigs/d): 3.3 (1.4–7.9) | Age, social factors |
| Mills et al. (1992) | UK | Europe | Case-Control | 216 | Smoking habits pre-onset | PsO (including PPP distribution) | OR (Pre-onset): 3.75 (1.68–9.47) | Age, sex, residence area |
| Naldi et al. (1992) | Italy | Europe | Multicentre Case-Control | 482 | Smoking status; intensity; alcohol | Incident PsO (newly diagnosed) | Adjusted OR (≥15 cigs/d): 2.1 | Education, family hx, sex |
| Poikolainen (1990) | Finland | Europe | Case-Control | 429 | Alcohol (g/day); type of beverage | Clinically diagnosed PsO (Men) | OR (100 g/day): 2.2 (1.3–3.9) | Age, smoking, SES |
| Huo et al. (2024) | USA | North America | Retrospective Cohort | 148,092 | Smoking history (ICD codes) | Incident PsA (PsO Smokers vs NS) | HR: Direction reported | PSM for comorbidities |
| Li W et al. (2023) | USA | North America | Prospective Cohort | 106,844 | Smoking status/cessation; Alcohol | Incident PsO (Medicare claims) | HR (≥20 pack-yrs): 1.53 | Ethnicity, income, BMI |
| Wu et al. (2015) | USA | North America | Prospective Cohort | 82,672 | Alcohol (g/day); Cumulative FFQ | Incident PsA (PASE questionnaire) | HR (≥30 g/d): 1.63 (1.00–2.67) | BMI, smoking, activity |
| Li W et al. (2012 AJE) | USA | North America | Pooled Cohort | 185,836 | Smoking dose (cigs/d, pack-yrs) | Incident physician-diagnosed PsO | RR (Current): 1.94 (1.64–2.28) | BMI, alcohol, activity |
| Li W et al. (2012 ARD) | USA | North America | Prospective Cohort | 94,874 | Smoking status and dose | Incident clinician-diagnosed PsA | RR (≥25 cigs/d): 2.11 (1.26–3.53) | Age, BMI, alcohol |
| Qureshi et al. (2010) | USA | North America | Prospective Cohort | 82,869 | Total alcohol (drinks/wk); subtypes | Incident PsO (Confirmed by PST) | RR (≥2.3 drinks/wk): 1.72 | Smoking, BMI, folate |
| Setty et al. (2007) | USA | North America | Prospective Cohort | 78,532 | Smoking intensity; pk-yrs; cessation | Incident physician-diagnosed PsO | RR (Current): 1.78 (1.46–2.16) | BMI, alcohol, age |

Abbreviations: PsO, Psoriasis; PsV, Psoriasis Vulgaris; PsA, Psoriatic Arthritis; PPP, Palmoplantar Pustulosis; GPP, Generalized Pustular Psoriasis.HR, Hazard Ratio; RR, Relative Risk; OR, Odds Ratio; aHR, Adjusted Hazard Ratio; CI, Confidence Interval. MR, Mendelian Randomization; NOS, Newcastle-Ottawa Scale.BMI, Body Mass Index; SES, Socioeconomic Status; ETS, Environmental Tobacco Smoke. NHIS, National Health Insurance Service (Korea); CPRD, Clinical Practice Research Datalink (UK); NHS, Nurses’ Health Study (USA).

Notes: 1. Selection Criteria: For case-control studies, incident cases (newly diagnosed within 1-2 years) were prioritized to minimize recall bias. 2. Alcohol Standardization: Alcohol consumption metrics were standardized to grams per day (g/day) based on standard drink definitions provided in the primary studies. 3. Adjustments: "Key Adjustments" list the core covariates used in the most fully adjusted model reported by the authors.

**Supplementary Table S3. Quality assessment of included studies 1**

| Study | Year | Country/Region | Design | Selection  (Max 4) | Comparability  (Max 2) | Outcome / Exposure  (Max 3) | Total Score  (Max 9) |
| --- | --- | --- | --- | --- | --- | --- | --- |
| Ashkevari et al. | 2011 | Iran | Case-Control | ★★ | ★★ | ★★★ | 7 |
| Dai et al. | 2019 | Taiwan | Cohort | ★★★★ | ★★ | ★★★ | 9 |
| Goto et al. | 2021 | Japan | Cohort | ★★★★ | ★★ | ★★★ | 9 |
| Green et al. | 2020 | UK | Cohort | ★★★★ | ★★ | ★★★ | 9 |
| Huerta et al. | 2007 | UK | Cohort | ★★★★ | ★★ | ★★★ | 9 |
| Huo et al. | 2024 | Global (USA based) | Cohort | ★★★★ | ★★ | ★★★ | 9 |
| Jankovic et al. | 2009 | Serbia/Montenegro | Case-Control | ★★★★ | ★★ | ★★ | 8 |
| Jordan et al. | 2024 | Denmark | Cohort | ★★★★ | ★★ | ★★★ | 9 |
| Kim et al. | 2024 | South Korea | Cohort | ★★★★ | ★★ | ★★★ | 9 |
| Lee et al. | 2017 | South Korea | Cohort | ★★★★ | ★★ | ★★★ | 9 |
| Li W et al. (ARD) | 2012 | USA | Cohort | ★★★★ | ★★ | ★★★ | 9 |
| Li W et al. (AJE) | 2012 | USA | Cohort | ★★★★ | ★★ | ★★★ | 9 |
| Li et al. | 2023 | USA | Cohort | ★★★★ | ★★ | ★★★ | 9 |
| Lønnberg et al. | 2016 | Denmark | Case-Control | ★★★★ | ★★ | ★★★ | 9 |
| Mills et al. | 1992 | UK | Case-Control | ★★★★ | ★ | ★★★ | 8 |
| Naldi et al. | 1992 | Italy | Case-Control | ★★★★ | ★★ | ★★★ | 9 |
| Naldi et al. | 1999 | Italy | Case-Control | ★★★★ | ★★ | ★★★ | 9 |
| Naldi et al. | 2005 | Italy | Case-Control | ★★★★ | ★★ | ★★★ | 9 |
| Näslund-Koch et al. | 2023 | Denmark | Cohort | ★★★★ | ★★ | ★★★ | 9 |
| Poikolainen et al. | 1990 | Finland | Case-Control | ★★★★ | ★★ | ★★★ | 9 |
| Poikolainen et al. | 1994 | Finland | Case-Control | ★★★★ | ★★ | ★★★ | 9 |
| Quan et al. | 2014 | China | Case-Control | ★★★★ | ★★ | ★★ | 8 |
| Qureshi et al. | 2010 | USA | Cohort | ★★★★ | ★★ | ★★★ | 9 |
| Setty et al. | 2007 | USA | Cohort | ★★★★ | ★★ | ★★★ | 9 |
| Taniguchi et al. | 2024 | Japan | Cohort | ★★★★ | ★★ | ★★★ | 9 |
| Wang et al. | 2021 | China | Case-Control | ★★★★ | ★★ | ★★ | 8 |
| Wolk et al. | 2009 | Sweden | Case-Control | ★★★★ | ★★ | ★★★ | 9 |
| Wu et al. | 2015 | USA | Cohort | ★★★★ | ★★ | ★★★ | 9 |
| Zhang et al. | 2002 | China | Case-Control | ★★ | ★ | ★★★ | 6 |
| Zheng et al. | 2004 | China | Case-Control | ★★ | ★ | ★★★ | 6 |

Non-randomized studies were assessed for methodological quality using the Newcastle–Ottawa Scale (NOS). The scale evaluates studies across three domains: selection, comparability, and outcome/exposure. In the selection and outcome/exposure domains, each item can be awarded a maximum of one star, while up to two stars can be assigned for comparability. Overall study quality was classified according to total NOS scores as follows: 0–3 stars indicating low quality, 4–6 stars indicating moderate quality, and 7–9 stars indicating high quality.

Li W et al. (ARD, 2012), published in Annals of the Rheumatic Diseases, focused on psoriatic arthritis, whereas Li W et al. (AJE, 2012), published in the American Journal of Epidemiology, focused on psoriasis.

**Supplementary Table S4. Exposure assignment and standardization methods used in dose–response analyses**

**a. Smoking intensity (cigarettes/day) 1**

| Study | Exposure metric | Original exposure category | Assigned dose | Unit | Assignment rule |
| --- | --- | --- | --- | --- | --- |
| Li W (2012 AJE) | Smoking intensity | 1–14 cigs/day | 7.5 | cigs/day | Midpoint of category |
| Li W (2012 AJE) | Smoking intensity | 15–24 cigs/day | 19.5 | cigs/day | Midpoint of category |
| Li W (2012 AJE) | Smoking intensity | ≥25 cigs/day | 30 | cigs/day | Open-ended upper: 1.2 × lower bound |
| Setty (2007) | Smoking intensity | 1–14 cigs/day | 7.5 | cigs/day | Midpoint of category |
| Setty (2007) | Smoking intensity | 15–24 cigs/day | 19.5 | cigs/day | Midpoint of category |
| Setty (2007) | Smoking intensity | ≥25 cigs/day | 30 | cigs/day | Open-ended upper: 1.2 × lower bound |
| Lee EJ (2017) | Smoking intensity | <10 cigs/day | 5 | cigs/day | Lower open-ended: midpoint of 0–upper bound |
| Lee EJ (2017) | Smoking intensity | 10–20 cigs/day | 15 | cigs/day | Midpoint of category |
| Lee EJ (2017) | Smoking intensity | 20–40 cigs/day | 30 | cigs/day | Midpoint of category |
| Lee EJ (2017) | Smoking intensity | ≥40 cigs/day | 48 | cigs/day | Open-ended upper: 1.2 × lower bound |
| Naldi (2005) | Smoking intensity | 1–10 cigs/day | 5.5 | cigs/day | Midpoint of category |
| Naldi (2005) | Smoking intensity | 11–20 cigs/day | 15.5 | cigs/day | Midpoint of category |
| Naldi (2005) | Smoking intensity | ≥21 cigs/day | 25.2 | cigs/day | Open-ended upper: 1.2 × lower bound |
| Naldi (1999) | Smoking intensity | ≤15 cigs/day | 7.5 | cigs/day | Lower open-ended: midpoint of 0–upper bound |
| Naldi (1999) | Smoking intensity | 16–24 cigs/day | 20 | cigs/day | Midpoint of category |
| Naldi (1999) | Smoking intensity | ≥25 cigs/day | 30 | cigs/day | Open-ended upper: 1.2 × lower bound |

b. Alcohol intake (grams of ethanol/day)

| Study | Exposure metric | Original exposure category | Assigned dose | Unit | Assignment rule |
| --- | --- | --- | --- | --- | --- |
| Wu (2015) | Alcohol intake | 0 g/day (Ref) | 0 | g/day | Reference category |
| Wu (2015) | Alcohol intake | 0.1–4.9 g/day | 2.5 | g/day | Midpoint of category |
| Wu (2015) | Alcohol intake | 5.0–9.9 g/day | 7.5 | g/day | Midpoint of category |
| Wu (2015) | Alcohol intake | 10.0–14.9 g/day | 12.5 | g/day | Midpoint of category |
| Wu (2015) | Alcohol intake | 15.0–29.9 g/day | 22.5 | g/day | Midpoint of category |
| Wu (2015) | Alcohol intake | ≥30.0 g/day | 36 | g/day | Open-ended upper: 1.2 × lower bound |
| Poikolainen (1990) | Alcohol intake | 0 g/day (Ref) | 0 | g/day | Reference category |
| Poikolainen (1990) | Alcohol intake | 1–49 g/day | 25 | g/day | Midpoint of category |
| Poikolainen (1990) | Alcohol intake | 50–99 g/day | 74.5 | g/day | Midpoint of category |
| Poikolainen (1990) | Alcohol intake | ≥100 g/day | 120 | g/day | Open-ended upper: 1.2 × lower bound |

**Footnote:**

Exposure levels were assigned using predefined and consistent rules for dose–response analyses. For closed exposure intervals, the midpoint of the category was used. For lower open-ended categories, the midpoint between zero and the upper boundary was assigned. For upper open-ended categories, exposure was estimated as 1.2 times the lower boundary. All assignments were based on exposure definitions reported in the original studies and were applied consistently to ensure comparability across analyses.

**Supplementary Table S5. PRISMA 2020 Checklist 1**

Preferred Reporting Items for Systematic Reviews and Meta-Analyses (PRISMA) 2020

| **Section** | **Item** | **PRISMA 2020 checklist item** | **Location reported in manuscript** |
| --- | --- | --- | --- |
| Title | 1 | Identify the report as a systematic review | Title |
| Abstract | 2 | Structured summary following PRISMA 2020 for Abstracts | Abstract |
| Introduction | 3 | Describe the rationale for the review | Introduction |
| Introduction | 4 | Provide an explicit statement of the objective(s) | Introduction |
| Methods | 5 | Specify inclusion and exclusion criteria | Section 2.3 (Eligibility criteria) |
| Methods | 6 | Specify information sources and search strategy | Section 2.2; Supplementary Table S1 |
| Methods | 7 | Describe the process for selecting studies | Section 2.4; Figure 1 (PRISMA flow diagram) |
| Methods | 8 | Describe the data collection process | Section 2.4 |
| Methods | 9 | List and define all outcomes | Section 2.4 |
| Methods | 10 | Describe methods for assessing risk of bias in included studies | Section 2.5; Supplementary Table S3 |
| Methods | 11 | Specify effect measures used (e.g., RR, OR, HR) | Section 2.7 |
| Methods | 12 | Describe synthesis methods | Section 2.7 |
| Methods | 13 | Describe methods for assessing heterogeneity | Section 2.7 |
| Methods | 14 | Describe methods for assessing reporting bias | Section 2.7 |
| Methods | 15 | Describe methods for sensitivity analyses | Section 2.7 |
| Results | 16 | Describe results of the study selection process | Section 3.1; Figure 1 |
| Results | 17 | Cite included studies and present their characteristics | Section 3.1; Supplementary Table S2 |
| Results | 18 | Present risk of bias assessments | Section 3.1; Supplementary Table S3 |
| Results | 19 | Present results of individual studies | Figures 2–6; Supplementary Figures |
| Results | 20 | Present results of all syntheses | Sections 3.2–3.4 |
| Results | 21 | Present assessments of heterogeneity | Sections 3.2–3.4 |
| Results | 22 | Present assessments of reporting bias | Sections 3.3.4, 3.4.2; Supplementary Figures |
| Results | 23 | Present results of additional analyses (dose–response, RCS, subgroup analyses) | Sections 3.2.4, 3.4.4; Supplementary Figures; Supplementary Table S4; Supplementary Table S6 |
| Discussion | 24 | Provide a general interpretation of the results | Section 4.1 |
| Discussion | 25 | Discuss limitations of the evidence | Section 4.6 |
| Discussion | 26 | Discuss implications for practice and future research | Section 4.4-4.6 and 5 |
| Other information | 27 | Report sources of funding and conflicts of interest | Declarations |

**Supplementary Table S6. Predicted Relative Risks of Psoriasis by Cumulative Smoking Exposure 1**

| Cumulative Exposure (Pack-Years) | Relative Risk (RR) | 95% Confidence Interval (CI) |
| --- | --- | --- |
| 10 Pack-Years | 1.25 | 1.05 – 1.55 |
| 20 Pack-Years | 1.52 | 1.20 – 1.95 |
| 30 Pack-Years | 1.7 | 1.35 – 2.15 |
| 40 Pack-Years | 1.75 | 1.45 – 2.05 |

**Note:** RR, relative risk; CI, confidence interval. Predicted values were derived from the random-effects dose–response meta-analysis using a restricted cubic spline (RCS) model (see Figure 3). The reference group was defined as never smokers (RR = 1.00).

**Supplementary Table S7. Detailed comparison of previous meta-analyses and the present study**

| **Study** | **Publication year** | **Exposure** | **Search end date** | **Included designs** | **No. of included studies** | **Dose–response analysis** | **Smoking cessation analysis** | **Subtype-specific outcomes** | **Main limitations** | **Added value relative to previous reviews** |
| --- | --- | --- | --- | --- | --- | --- | --- | --- | --- | --- |
| Armstrong | 2013 | Smoking | 15 Jun 2013 | Prevalence studies and incidence studies | 25 prevalence + 3 incidence studies | No formal pooled dose–response meta-analysis; only a possible dose effect was noted | No | No formal pooled subtype analysis | Focused mainly on smoking prevalence; limited incident evidence; no pooled multi-metric dose–response synthesis | Established smoking as a relevant exposure in psoriasis but did not provide the broader updated framework used in the present study |
| Zhou | 2020 | Smoking | 12 Apr 2020 | Cohort and case–control studies; also treatment-response studies | 34 studies for smoking risk; 16 for treatment efficacy | No formal pooled dose–response meta-analysis; heavier smoking was discussed descriptively | Limited / unclear | PPP and PsA identified but not formally pooled in the main smoking-risk synthesis | Smoking-focused only; mixed aims (risk and treatment efficacy); subtype and cessation evidence remained limited | Updated smoking evidence but did not integrate alcohol, exploratory cessation analyses, or multi-metric smoking dose–response analyses |
| Brenaut | 2013 | Alcohol | Up to Dec 2011 | Heterogeneous observational studies | 28 selected articles; 5 addressed alcohol as a psoriasis risk factor | No | No | No | Could not perform meta-analysis because alcohol exposure definitions were too heterogeneous | Important qualitative review, but not a pooled quantitative synthesis of alcohol-related psoriasis risk |
| Zhu | 2012 | Alcohol | Up to 2012 | Case–control studies only | 15 | No formal dose–response meta-analysis | No | No | Restricted to case–control studies; focused on prevalent psoriasis; limited database/language scope | Provided an early pooled alcohol estimate but without an incident-focused or subtype-specific framework |
| Present study | 2026 | Smoking and alcohol | 18 Dec 2025 | Cohort, case–control, nested case–control, twin studies | 30 | Yes; smoking across pack-years, duration, and cigarettes/day; alcohol-related dose–response also examined | Yes, exploratory | Yes; PPP and PsA considered, with progression-related analyses separated from primary incidence synthesis | Residual heterogeneity; mixed effect measures; limited evidence for some subtype-specific and cessation-related analyses; no trial-registry/grey-literature search | Updated integrated synthesis of smoking and alcohol; emphasis on incident/newly diagnosed psoriasis where possible; multi-metric smoking dose–response analysis; exploratory smoking cessation analysis; subtype-specific evaluation; bias correction for alcohol using trim-and-fill |

Legend: This table summarizes key differences between previous reviews and the present study, including search period, exposure scope, eligible study designs, number of included studies, dose–response analysis, smoking cessation analysis, subtype-specific outcomes, major limitations, and the added value of the current review.

**Supplementary Table S8. Stratified analyses by effect measure type for the association between smoking and incident psoriasis**

| **Exposure** | **Outcome** | **Effect measure** | **No. of studies** | **Pooled estimate** | **95% CI** | **I² (%)** | **Effect-measure-specific subgroup analysis** |
| --- | --- | --- | --- | --- | --- | --- | --- |
| Smoking | Incident psoriasis | Effect-measure-specific subgroup analysis | 6 | 1.84 | 1.52–2.22 | 71.8 | Primary analysis |
| Smoking | Incident psoriasis | Single study; not pooled | 1 | 1.14 | 1.13–1.15 | NA | Single study |
| Smoking | Incident psoriasis | Effect-measure-specific subgroup analysis | 5 | 1.66 | 1.31–2.10 | 91.4 | Primary analysis |

Legend: This table shows the stratified analyses of the association between smoking and incident psoriasis by effect measure type (OR-only, RR-only, HR-only). The pooled estimates and corresponding 95% confidence intervals (CIs) are presented along with the heterogeneity statistics (I²).

Supplementary Table S9. Covariate adjustment for mutual smoking/alcohol confounding and other key covariates

| **Study** | **Design / population** | **Alcohol evaluated as exposure?** | **Smoking adjusted in alcohol analysis?** | **Alcohol adjusted in smoking analysis?** | **BMI adjusted?** | **SES / education adjusted?** | **Cardiometabolic factors adjusted?** | **Other reported covariates / reviewer-relevant notes** |
| --- | --- | --- | --- | --- | --- | --- | --- | --- |
| Zheng et al. (2004) | Case-control; China; 189 psoriasis patients and 333 healthy controls; HLA-DQA1*0201 interaction analysis. | Yes; alcohol consumption was assessed and analyzed with sex and HLA-DQA1*0201 genotype. | No / not reported; alcohol and smoking were analyzed, but mutual adjustment was not clearly reported. | No / not reported. | No / not reported. | No / not reported. | No / not reported. | Early genetic-environment study; alcohol and smoking co-occurrence was not adequately controlled. |
| Li et al. (2012, PsA) | Prospective cohort; Nurses' Health Study II; 94,874 women; incident PsA outcome. | No; main exposure was smoking. | N/A. | Yes; smoking model adjusted for alcohol drinking categories. | Yes. | No / not clearly reported. | No in the displayed main smoking model. | Other adjustments included age and vigorous physical activity. |
| Wu et al. (2015) | Prospective cohort; Nurses' Health Study II; 82,672 women; incident PsA outcome. | Yes; cumulative average, updated, and baseline alcohol intake were analyzed. | Yes; alcohol model adjusted for smoking status and cigarettes/day categories. | N/A; alcohol was the main exposure. | Yes. | No / not clearly reported. | Yes; cardiovascular disease, type 2 diabetes, hypertension, and hypercholesterolemia were included in fully adjusted models. | Other adjustments included physical activity, acetaminophen use, NSAID use, menopausal status/postmenopausal hormone use, multivitamin use, and folate intake. |
| Green et al. (2020) | Population-based cohort; UK CPRD; 90,189 incident psoriasis cases; PsA development outcome. | Yes; alcohol status was categorized as non-drinker, moderate drinker, heavy drinker, and ex-drinker. | Unclear; the article considered smoking, alcohol, and BMI, but the alcohol-model footnote did not clearly list smoking adjustment. | N/A; alcohol was one of the lifestyle exposures. | Yes; alcohol model adjusted for BMI category. | No / not clearly reported. | Partly; diabetes was considered among potential confounders, but not clearly listed in the final alcohol model footnote. | Alcohol model adjusted for age, psoriasis duration, psoriasis severity, and BMI category. Interpret alcohol findings cautiously. |
| Wang et al. (2021) | Case-control; China; 171 PsA, 342 psoriasis, and 1026 healthy controls matched by age and sex. | Yes; alcohol drinking was analyzed as a behavioral factor. | Yes; adjusted models included smoking and alcohol drinking. | Yes; smoking model adjusted for alcohol drinking. | Yes for PsA model; not listed in the psoriasis-model footnote when BMI was treated as an analyzed factor. | Yes; education level adjusted. | Yes; comorbidities included hypertension, dyslipidemia, diabetes, and/or coronary artery disease depending on model. | Useful example of mutual behavioral adjustment, although exposure categories were broad. |
| Naslund-Koch et al. (2023) | Prospective observational and Mendelian randomization analysis; Copenhagen General Population Study; 105,912 individuals; moderate-to-severe psoriasis outcome. | No; main exposure was smoking. | N/A. | Yes; observational smoking model adjusted for high alcohol consumption. | Yes. | Yes; low education level adjusted. | Yes; hypertension, dyslipidemia, and type 2 diabetes adjusted. | Other adjustments included age, sex, and low physical activity. |
| Huo et al. (2024) | Retrospective cohort; TriNetX database; psoriasis to PsA transition; smoking history and type 2 diabetes evaluated. | No; alcohol was not the main exposure. | N/A. | Not a direct smoking/alcohol mutual-adjustment model; alcohol-related disorders were included among covariates. | No / not clearly reported. | Yes; socioeconomic/psychosocial variables were included among covariates. | Yes; hypertensive diseases, hyperlipidemia, heart failure, chronic kidney disease, liver diseases, and type 2 diabetes were considered. | Useful for documenting whether alcohol-related disorders were considered when evaluating smoking and PsA transition. |
| Jankovic et al. (2009) | Case-control; Montenegro; 110 psoriasis outpatients and 200 dermatologic controls. | Yes; alcohol consumption analyzed as a risk factor. | Unclear / likely limited; multivariable model adjusted for sex, age, BMI, and disease duration, but mutual smoking adjustment was not clearly specified for alcohol. | Unclear / not clearly reported. | Yes. | No / not clearly reported. | No / not clearly reported. | Environmental tobacco smoke and stressful life events were evaluated. Residual smoking-alcohol confounding remains possible. |
| Zhang et al. (2002) | Case-control; China; 789 psoriasis patients and 789 healthy controls. | Yes; alcohol drinking assessed and compared between cases and controls. | No / not reported; mutually adjusted multivariable model was not reported. | No / not reported. | No / not reported. | Matching/description included occupation, education, and residence, but multivariable SES adjustment was not reported. | No / not reported. | Early case-control evidence; especially vulnerable to smoking-alcohol co-occurrence. |
| Taniguchi et al. (2024) | Retrospective cohort; JMDC claims/checkup database; 717,472 individuals; incident PPP outcome. | Yes; daily alcohol consumption included in multivariable Cox model. | Yes; model included smoking status and daily alcohol consumption simultaneously. | Yes; model included daily alcohol consumption when estimating smoking association. | Yes. | No / not reported. | Yes; diabetes mellitus, hyperlipidemia, and hypertension included. | Other adjustments included sex, age, chronic tonsillitis, periodontal disease, and smoking-by-sex interaction. |
| Quan et al. (2014) | Hospital-based case-control; China; 345 psoriasis vulgaris cases and 315 controls; BDNF rs6265 interaction analysis. | Yes; alcohol consumption was coded as drinker/non-drinker. | Yes; logistic regression adjusted for smoking, alcohol consumption, and BMI, with age and sex. | Yes; smoking was analyzed in models also including alcohol consumption. | Yes. | No / not reported. | No / not reported. | Other adjustments included age, sex, and BDNF genotype-related interaction terms; alcohol and smoking categories were broad. |
| Wolk et al. (2009) | Population-based case-control; Sweden; 373 first-onset plaque psoriasis cases and matched controls. | Yes; alcohol consumption was analyzed as monthly drinks. | Yes; onset model included BMI, weight gain, alcohol, and smoking, with matched variables. | Yes; smoking estimate was from the same mutually adjusted model. | Yes. | Partly; matched on postal code and economic status was assessed, but SES was not clearly included in the main onset model. | No / not reported. | Other adjustments included age, sex, postal code, and weight gain; alcohol association was positive among men but not women. |
| Naldi et al. (1992) | Multicenter case-control; Italy; 215 newly diagnosed psoriasis cases and 267 dermatologic controls. | Yes; alcohol consumption analyzed as drinks/day. | Yes; MLR model included smoking habits and alcohol consumption. | Yes; same MLR model included alcohol and smoking. | No / not reported. | Yes; education or occupational status and marital status were included. | No / not reported. | Other adjustments included age, sex, family history in parents/siblings, coffee consumption, and number of siblings when indicated. |
| Kim et al. (2024) | Retrospective cohort; Korea; 5,784,973 participants; incident PsV, PPP, and GPP outcomes. | No; main exposure was smoking cessation status/pack-years. | N/A. | Yes; smoking-cessation models adjusted for alcohol. | Yes. | Yes; income adjusted. | No / not clearly reported in the available summary. | Other adjustments included age; row mainly supports that some smoking analyses controlled for alcohol. |
| Poikolainen et al. (1990) | Case-control; Finland; men aged 19-50; 144 psoriasis patients and 285 dermatologic controls. | Yes; alcohol intake was the primary exposure. | Yes; logistic regression considered smoking along with alcohol, age, marital state, social group, and coffee consumption. | Yes / model considered alcohol when examining smoking as a possible factor. | No / not reported. | Yes; marital state and social group considered. | No / not reported. | Male-only study; alcohol association remained while smoking was not significant in the logistic regression. |
| Li et al. (2023, WHI) | Prospective cohort; Women's Health Initiative; 106,844 postmenopausal women; incident psoriasis outcome. | Yes; alcohol status, frequency, and subtype were analyzed. | Yes; full model adjusted for cigarette smoking, and additional analyses stratified by smoking status. | Partly; smoking-related analyses were assessed with alcohol habits/stratification rather than a simple uniform adjustment statement. | Yes. | Yes; income and ethnicity were adjusted. | No / not as main cardiometabolic covariates; history of non-melanoma skin cancer was adjusted. | Other adjustments/strata included age and WHI component randomization status. Alcohol association disappeared after smoking adjustment. |
| Jordan et al. (2024) | Prospective observational plus Mendelian randomization; Danish general population; 102,655 adults; incident psoriasis outcome. | Yes; alcohol consumption was the main exposure. | Yes; fully adjusted observational model included smoking status. | N/A; smoking was not the main exposure. | Yes. | Yes; low education and low income adjusted. | Partly; diabetes was included in the full model. | Other adjustments included study cohort, year of inclusion, sex, age, poor mental health, and physical inactivity. MR analysis addressed confounding in a complementary way. |
| Li et al. (2012, psoriasis) | Prospective combined analysis; NHS, NHS II, and HPFS; 185,836 participants; incident psoriasis outcome. | No; main exposure was smoking. | N/A. | Yes; smoking models adjusted for alcohol intake. | Yes. | No / not clearly reported in main smoking model. | Partly; chronic diseases were described but not uniformly listed in the main smoking model. | Other adjustments included age and cohort-specific covariates such as physical activity; passive smoke models adjusted for alcohol and self-smoking status. |
| Qureshi et al. (2010) | Prospective cohort; Nurses' Health Study II; 82,869 women; incident psoriasis outcome. | Yes; total alcohol and beverage type were analyzed. | Yes; multivariate alcohol models adjusted for smoking. | N/A; alcohol was the main exposure. | Yes. | No / not clearly reported. | No / not reported. | Other adjustments included age, physical activity, and dietary folate equivalents; strong relevance to reviewer concern because smoking distribution differed by alcohol intake. |
| Huerta et al. (2007) | Prospective cohort with nested case-control analysis; UK GPRD; 3994 psoriasis cases and 10,000 controls. | Alcohol was recorded, but the main reported behavioral risk factor was smoking. | No clear alcohol model was reported; not sufficient to judge smoking adjustment for alcohol analysis. | No / not clearly reported for main smoking estimate. | Yes; BMI included in multivariable regression. | No / not clearly reported. | Comorbidities were evaluated as risk factors, but not clearly listed as adjustment covariates for smoking/alcohol. | Model included frequency-matching variables, number of GP visits in the previous year, smoking, and BMI. This row is less informative for alcohol-specific confounding. |
| Goto et al. (2021) | Population-based cohort; Japan; 487,835 individuals aged 40-107 years; late-onset incident psoriasis outcome. | No; alcohol consumption was not reported as a main exposure in the final multivariable risk model, although liver-related GGT was included. | N/A. | No / not clearly reported; smoking model did not clearly include alcohol consumption as a covariate. | Yes; BMI was included in multivariable Cox models. | No / not reported. | Partly; GGT was included and cardiometabolic variables were discussed/available, but diabetes and hypertension were not retained as final adjusted covariates for the main risk model. | Other adjusted factors included age, sex, current smoking, low physical activity, and either BMI or weight gain since age 20. This row is mainly relevant to smoking rather than alcohol-specific confounding. |
| Naldi et al. (2005) | Case-control; Italy; 560 recent-onset psoriasis cases and 690 dermatologic controls. | Yes; alcohol consumption was included among candidate environmental risk factors. | Yes; multiple logistic regression included smoking and alcohol habits in the same model. | Yes; smoking estimates were adjusted for alcohol habits. | Yes. | Yes; education level was included. | No / not reported. | Other adjustments included sex, age, marital status, hospitalization, BMI, and stressful life events. The paper explicitly evaluated combined effects of smoking with alcohol, BMI, family history, and life events. |
| Poikolainen et al. (1994) | Case-control; Finland; women aged 18-50; 55 psoriasis patients and 108 dermatologic controls. | Yes; alcohol intake before and after disease onset was analyzed. | Yes; logistic regression considered alcohol and smoking together, along with marital status and social group. | Yes / same logistic regression framework considered alcohol when evaluating smoking. | No / not reported. | Yes; marital status and social group were considered. | No / not reported. | Female-only study. Before onset, psoriasis was significantly associated with smoking but not alcohol; after onset, alcohol, smoking, and negative life events were associated with psoriasis. |
| Dai et al. (2019) | Population-based cohort; Taiwan NHIS linked to NHIRD; 60,136 participants; incident psoriasis outcome. | Yes; alcohol consumption was analyzed as never, social, regular, or heavy drinking. | Yes; multivariable Cox models included smoking status when evaluating alcohol categories. | Yes; smoking models adjusted for alcohol consumption. | Yes. | Yes; marital status, educational level, and monthly household income were adjusted. | Yes; comorbidities were adjusted, including diabetes, hyperlipidemia, hypertension, COPD, chronic liver disease, chronic kidney disease, and related conditions. | Alcohol was not significantly associated with psoriasis after adjustment, whereas current smoking was associated with higher risk. Alcohol exposure was broad and did not quantify drinks consumed. |
| Setty et al. (2007) | Prospective cohort; Nurses' Health Study II; 78,532 women; incident psoriasis outcome. | No; main exposure was smoking status, intensity, duration, pack-years, cessation, and passive smoke. | N/A. | Yes; multivariate smoking models adjusted for alcohol intake categories. | Yes. | Partly; husband's education was evaluated but not retained as a major adjustment that changed estimates. | No / not reported. | Other adjustments included age. Reproductive factors and husband's education were tested and did not materially change smoking estimates. |
| Lonnberg et al. (2016) | Population-based twin study; Denmark; 34,781 twins aged 20-71 years; psoriasis and smoking history assessed by questionnaire. | No; main exposure was smoking and childhood environmental tobacco smoke. | N/A. | No / not reported; alcohol was discussed as a potential correlated factor but was not clearly included in the main smoking adjustment model. | No / not reported. | No / not reported. | No / not reported. | Multivariable model adjusted for age, sex, and childhood environmental tobacco smoke; twin-pair analyses addressed shared genetic/environmental confounding rather than alcohol confounding. |
| Lee et al. (2017) | Nationwide cohort; Korea National Health Insurance; 17,055,608 adults followed for 8 years; incident psoriasis outcome. | No; main exposure was smoking status, amount, and duration. | N/A. | Yes; multivariable incidence-rate analysis adjusted for alcohol consumption status. | Yes. | No / not clearly reported. | Yes; diabetes mellitus, hypertension, and dyslipidemia were adjusted. | Other adjustments included age, sex, and physical activity/exercise. Strong large-scale example of a smoking analysis controlling for alcohol. |
| Mills et al. (1992) | Case-control; UK; 108 psoriasis patients and matched community controls. | No; study focused on smoking habits. | N/A. | No / not reported; alcohol was not incorporated into the smoking analysis. | No / not reported. | Partly; cases and controls were matched by age, sex, and area of residence as a proxy for social class; social class distribution was examined. | No / not reported. | Early smoking-focused study with matched design. It provides limited information for smoking-alcohol mutual confounding. |
| Ashkevari et al. (2011) | Comparative case-control; Iran; 96 psoriasis vulgaris patients and 96 healthy controls matched by age, sex, and BMI. | No; alcohol users were excluded according to the study criteria rather than analyzed as an exposure. | N/A; alcohol consumption was excluded, so residual alcohol confounding was reduced by design but not estimated. | N/A / not applicable; alcohol users were excluded from the smoking comparison. | Yes; controls were matched by BMI and BMI >30 was excluded. | No / not reported. | No / not reported. | The study excluded alcohol use, major psychological stress, and BMI >30, making it a smoking-focused analysis after removing several confounders by design. |
| Naldi et al. (1999) | Multicenter case-control; Italy; 404 early-stage psoriasis cases and 616 dermatologic controls. | Yes; alcohol consumption was analyzed overall and by sex. | Yes; alcohol association was assessed after controlling for smoking habits and other potential confounders. | Yes; smoking model included alcohol consumption in the multiple logistic regression equations. | Yes. | Yes; occupation and marital status were included. | No / not reported. | Other adjustments included age, sex, calendar year at interview, family history in first-degree relatives, BMI, coffee consumption, smoking habits, and alcohol consumption. Alcohol association was mainly observed in men. |

**Abbreviations:** BMI, body mass index; COPD, chronic obstructive pulmonary disease; CPRD, Clinical Practice Research Datalink; ETS, environmental tobacco smoke; GGT, gamma-glutamyl transpeptidase; GPP, generalized pustular psoriasis; HPFS, Health Professionals Follow-up Study; MLR, multiple logistic regression; MR, Mendelian randomization; N/A, not applicable; NHS, Nurses Health Study; NHIRD, National Health Insurance Research Database; NHIS, National Health Interview Survey; NSAID, nonsteroidal anti-inflammatory drug; OR, odds ratio; PPP, palmoplantar pustulosis; PsA, psoriatic arthritis; PsV, psoriasis vulgaris; SES, socioeconomic status; WHI, Women Health Initiative.

**Supplementary Figures**

**Supplementary Figure S1. Association between smoking and incident psoriasis stratified by study design 1**

| a | b |
| --- | --- |
| 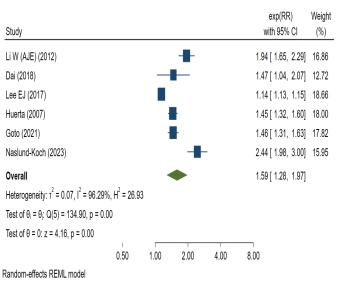 | 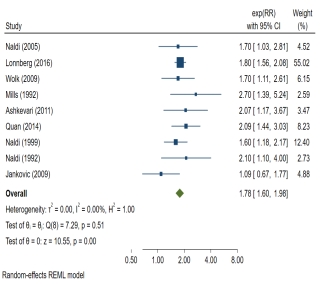 |

Legend: (a) Forest plot showing the association between smoking and the risk of incident psoriasis based on cohort studies only.

(b) Forest plot showing the association between smoking and the risk of incident psoriasis based on case–control studies only.

Study-specific relative risks (RRs) and 95% confidence intervals (CIs) were pooled using a random-effects model to assess the consistency of results across different study designs.

**Supplementary Figure S2. Study-level dose–response visualizations for smoking exposure and incident psoriasis risk1**

| a | b | c |
| --- | --- | --- |
| 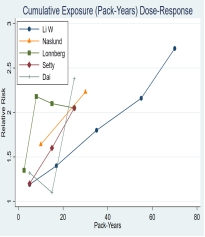 | 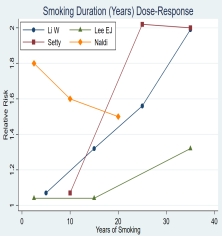 | 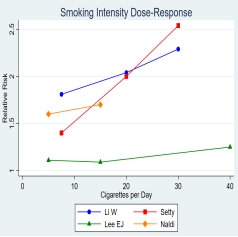 |

**Legend：**Descriptive restricted cubic spline curves are shown for (a) pack-years, (b) smoking duration, and (c) cigarettes per day, based on the available study-specific exposure data. These plots illustrate study-level exposure patterns and should not be interpreted as pooled meta-analytic dose–response estimates.

**Supplementary Figure S3. Funnel plot assessing publication bias for the association between smoking and psoriasis risk 1**

| 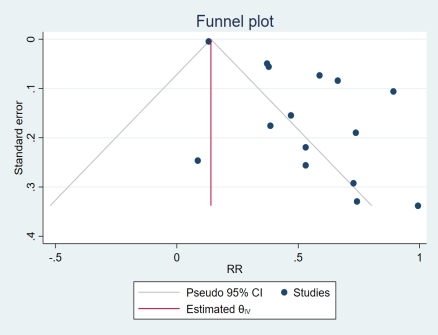 |
| --- |

Legend：Funnel plot with pseudo 95% confidence limits used to assess potential publication bias in studies evaluating the association between smoking and the risk of psoriasis.

**Supplementary Figure S4. Association between smoking and psoriasis-related outcomes including disease incidence and progression 1**

| 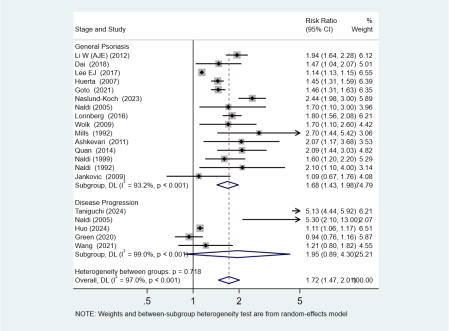 |
| --- |

Legend：Forest plot showing the association between smoking and psoriasis-related outcomes, including incident psoriasis and disease progression or related subtypes. Pooled estimates were calculated using a random-effects model. Owing to heterogeneity in outcome definitions, results should be interpreted with caution.

**Supplementary Figure S5. Smoking cessation and psoriasis risk relative to current smoking across clinical subtypes1**

| 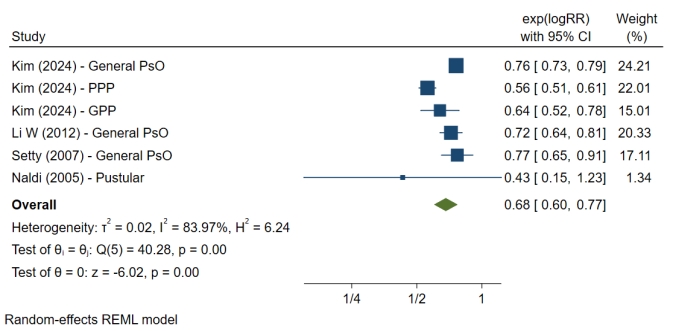 |
| --- |

Legend: Forest plot showing study-specific relative risks (RRs) and 95% confidence intervals (CIs) comparing former (quit/past/ex) smokers with current smokers, stratified by psoriasis clinical subtype where available. When a single study reported subtype-specific estimates, these were presented separately for descriptive purposes. Because of heterogeneity in exposure definitions, outcome classification, and the inclusion of multiple non-independent estimates from the same study, the pooled estimate derived from a random-effects model should be interpreted as exploratory rather than as a definitive quantitative summary.

**Supplementary Figure S6. Long-term trajectory of psoriasis risk following smoking cessation 1**

| 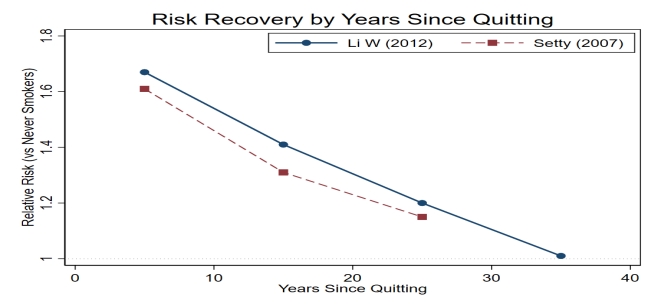 |
| --- |

Legend: Line plot showing changes in the relative risk of psoriasis among former smokers compared with never smokers according to years since smoking cessation, based on data from two cohort studies. The relative risk declines progressively with increasing time since quitting, suggesting a gradual recovery process. This analysis is descriptive and exploratory in nature and is intended to illustrate long-term trends in risk recovery rather than to provide a definitive estimate of the time required for risk normalization. Results are based on available study-specific data and should be considered hypothesis-generating rather than a precise estimate of risk normalization over time.

**Supplementary Figure S7. Funnel plot assessing publication bias for residual psoriasis risk in former smokers 1**

| 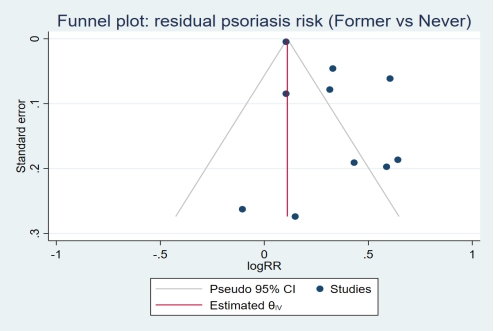 |
| --- |

Legend：Funnel plot with pseudo 95% confidence limits used to assess potential publication bias in studies evaluating residual psoriasis risk among former smokers.

**Supplementary Figure S8. Leave-one-out sensitivity analysis of residual psoriasis risk in former smokers 1**

| 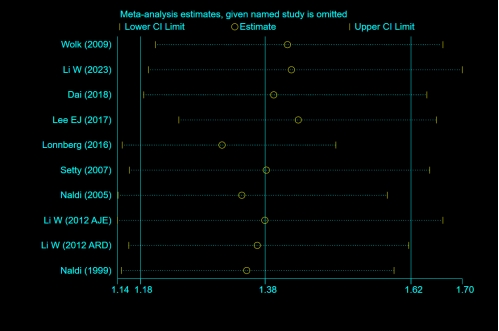 |
| --- |

Legend：Leave-one-out sensitivity analysis evaluating the robustness of the pooled estimate for residual psoriasis risk among former smokers compared with never smokers. The pooled estimate remained stable after sequential exclusion of individual studies, indicating that no single study disproportionately influenced the results.

**Supplementary Figure S9. Association between alcohol consumption and incident psoriasis based on OR-only studies**


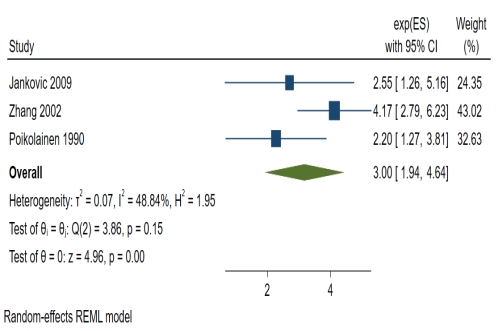


Legend：Forest plot showing the association between alcohol consumption and the risk of incident psoriasis based on studies reporting odds ratios (ORs) only. Study-specific effect estimates and 95% confidence intervals (CIs) were pooled using a random-effects model (restricted maximum likelihood, REML). The size of the squares represents the weight of each study, and the diamond indicates the overall pooled estimate. Heterogeneity was assessed using the I² statistic and Cochran’s Q test.

**Supplementary Figure S10. Dose–response relationship between alcohol intake and risk of psoriatic arthritis 1**

| 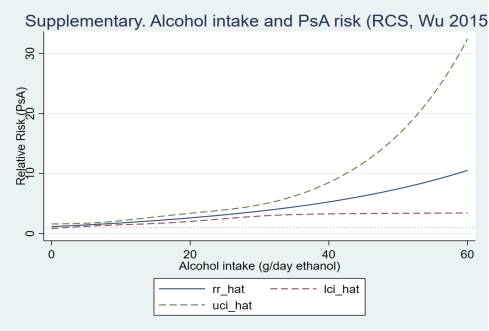 |
| --- |

**Legend:** Restricted cubic spline analysis illustrating the non-linear association between daily alcohol intake (grams/day) and psoriatic arthritis risk, based primarily on data from Wu et al. (2015). This figure is provided as exploratory single-study evidence and should not be interpreted as definitive pooled meta-analytic dose–response evidence.

**Supplementary Figure S11. Funnel plot assessing publication bias for the association between alcohol consumption and incident psoriasis1**

| 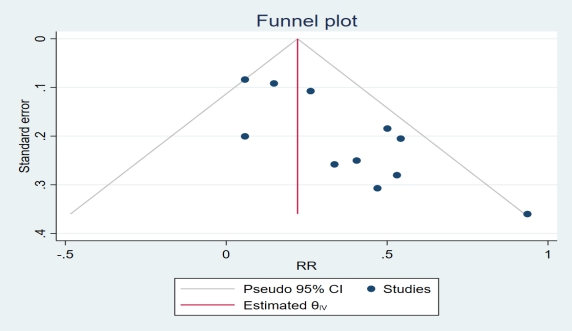 |
| --- |

Legend：Funnel plot with pseudo 95% confidence limits used to assess potential publication bias in studies evaluating the association between alcohol consumption and the risk of incident psoriasis.

**Supplementary Figure S12. Trim-and-fill analysis for the association between alcohol consumption and incident psoriasis1**

| 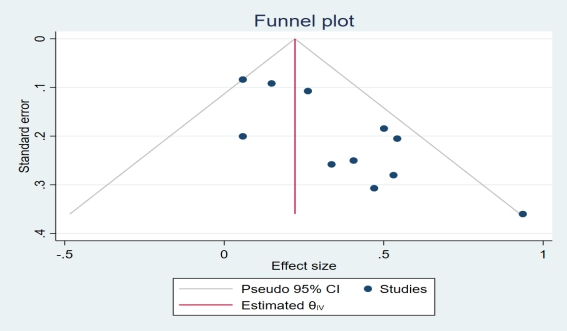 |
| --- |

Legend：Trim-and-fill analysis assessing the potential impact of publication bias on the association between alcohol consumption and the risk of incident psoriasis. The figure illustrates pooled estimates before and after adjustment for potentially missing studies.

**Supplementary Figure S13. Association between alcohol consumption and risk of psoriatic arthritis (secondary prevention analysis) 1**

| 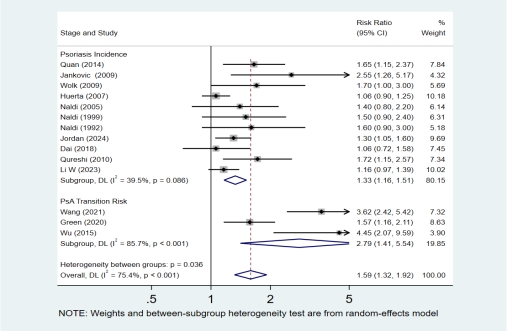 |
| --- |

Legend: Forest plot showing the pooled relative risk of transitioning to psoriatic arthritis among psoriasis patients with alcohol consumption compared with non-drinkers. Pooled estimates were calculated using a random-effects model due to significant heterogeneity ($I^2 = 85.7\%$). The diamond represents the overall pooled RR, and squares represent individual study estimates with 95% confidence intervals.

**Supplementary Figure S14. Stratified analysis of the association between smoking and incident psoriasis by effect measure type**


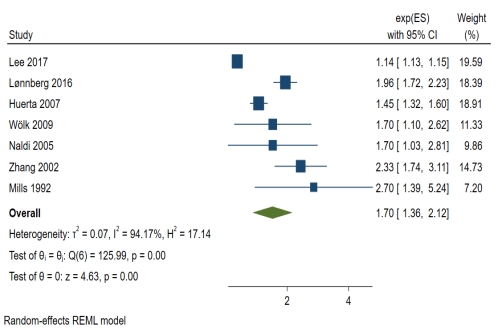


Legend: Forest plot showing the association between smoking and the risk of incident psoriasis after excluding studies reporting hazard ratios (HRs). Study-specific effect estimates and 95% confidence intervals (CIs) were pooled using a random-effects model (restricted maximum likelihood, REML). The size of the squares represents the weight of each study, and the diamond indicates the overall pooled estimate. Heterogeneity was assessed using the I² statistic and Cochran’s Q test. This analysis was conducted to assess the robustness of the primary findings given the conceptual differences between HRs and other effect measures.

**Supplementary Figure S15. Study-level dose–response visualization of daily alcohol intake and psoriasis-related outcomes1**


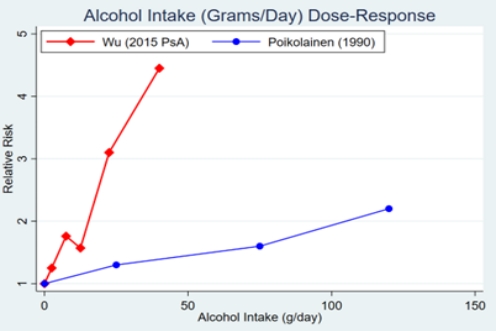


Legend: Descriptive restricted cubic spline curves illustrating the study-level relationship between daily alcohol intake (grams/day) and psoriasis-related outcomes based on the available study-specific exposure data. This figure is provided to show the pattern of alcohol-intake categories across contributing studies and should not be interpreted as definitive pooled meta-analytic dose–response evidence. Psoriatic arthritis (PsA)-specific alcohol dose–response evidence based primarily on Wu et al. (2015) is presented separately in Supplementary Figure S10.

Supplementary Figure S16. Sensitivity analysis excluding HR-based estimates for the association between smoking and incident psoriasis
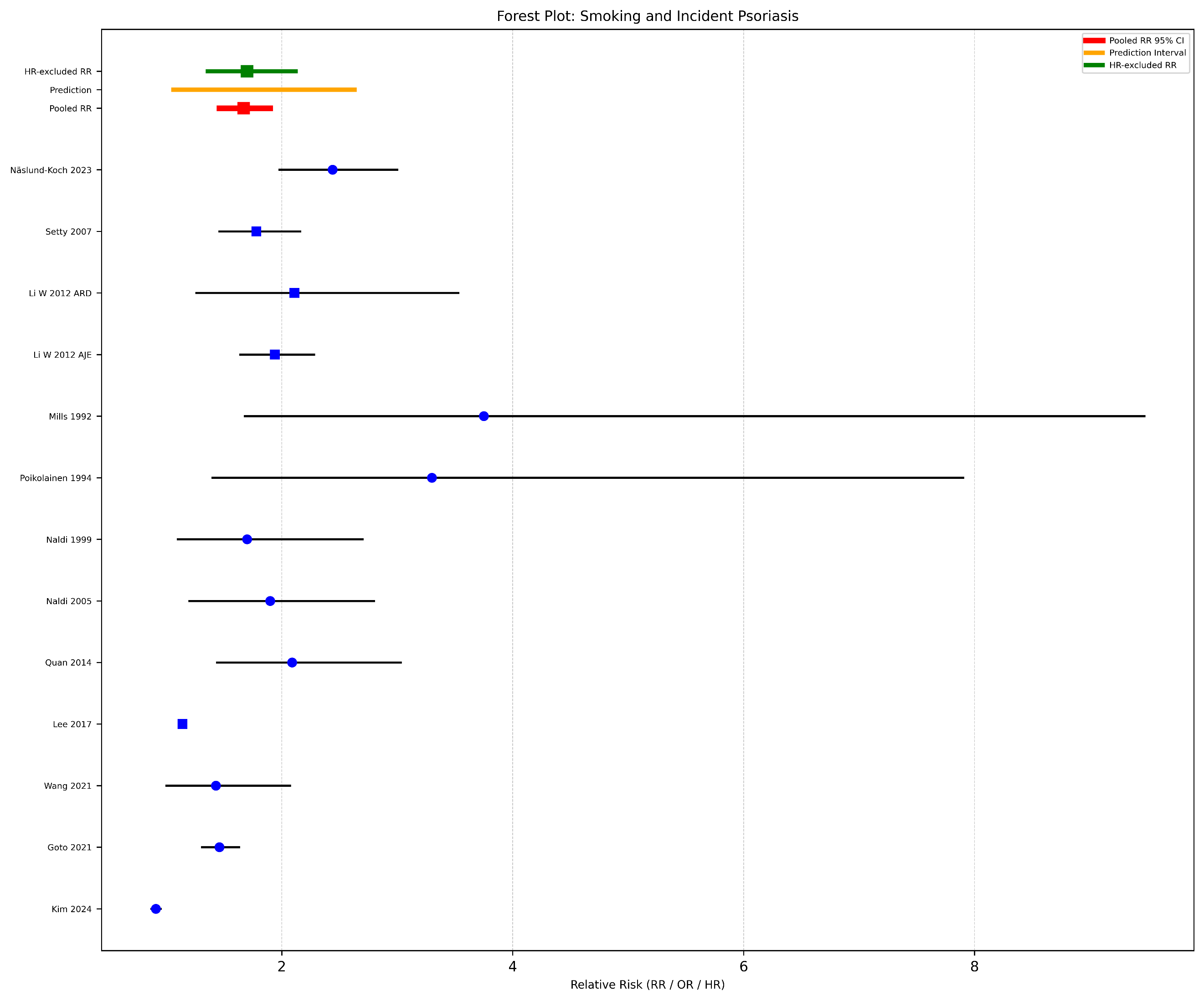


Legend: Sensitivity analysis excluding HR-based estimates for the association between smoking and incident psoriasis. Study-specific effect estimates are shown with corresponding 95% confidence intervals. The primary pooled estimate was RR = 1.67 (95% CI: 1.46–1.90), whereas the pooled estimate after excluding HR-based estimates was RR = 1.70 (95% CI: 1.36–2.12). The absolute difference between the two pooled point estimates was 0.03, indicating that exclusion of HR-based studies did not materially change the direction or approximate magnitude of the association. The prediction interval is also shown to reflect between-study uncertainty.
